# The influence of pregnancy on the pharmacokinetic properties of artemisinin combination therapy (ACT): a systematic review

**DOI:** 10.1186/s12936-016-1160-6

**Published:** 2016-02-18

**Authors:** Renée J. Burger, Benjamin J. Visser, Martin P. Grobusch, Michèle van Vugt

**Affiliations:** Division of Internal Medicine, Department of Infectious Diseases, Academic Medical Center, Center of Tropical Medicine and Travel Medicine, University of Amsterdam, Meibergdreef 9, PO Box 22700, 1100 DE Amsterdam, The Netherlands; Centre de Recherches de Médicales de Lambaréné (CERMEL), Albert Schweitzer Hospital, Lambaréné, Gabon

**Keywords:** Artemisinin combination therapy, ACT, Pregnancy, Pharmacokinetics, *Plasmodium falciparum*, *P. vivax*, *P. ovale*, *P. malariae*, *P. knowlesi*

## Abstract

**Background:**

Pregnancy has been reported to alter the pharmacokinetic properties of anti-malarial drugs, including the different components of artemisinin-based combination therapy (ACT). However, small sample sizes make it difficult to draw strong conclusions based on individual pharmacokinetic studies. The aim of this review is to summarize the evidence of the influence of pregnancy on the pharmacokinetic properties of different artemisinin-based combinations.

**Methods:**

A PROSPERO-registered systematic review to identify clinical trials that investigated the influence of pregnancy on the pharmacokinetic properties of different forms of ACT was conducted, following PRISMA guidelines. Without language restrictions, Medline/PubMed, Embase, Cochrane Central Register of Controlled Trials, Web of Science, LILACS, Biosis Previews and the African Index Medicus were searched for studies published up to November 2015. The following components of ACT that are currently recommend by the World Health Organization as first-line treatment of malaria in pregnancy were reviewed: artemisinin, artesunate, dihydroartemisinin, lumefantrine, amodiaquine, mefloquine, sulfadoxine, pyrimethamine, piperaquine, atovaquone and proguanil.

**Results:**

The literature search identified 121 reports, 27 original studies were included. 829 pregnant women were included in the analysis. Comparison of the available studies showed lower maximum concentrations (C_max_) and exposure (AUC) of dihydroartemisinin, the active metabolite of all artemisinin derivatives, after oral administration of artemether, artesunate and dihydroartemisinin in pregnant women. Low day 7 concentrations were commonly seen in lumefantrine studies, indicating a low exposure and possibly reduced efficacy. The influence of pregnancy on amodiaquine and piperaquine seemed not to be clinically relevant. Sulfadoxine plasma concentration was significantly reduced and clearance rates were higher in pregnancy, while pyrimethamine and mefloquine need more research as no general conclusion can be drawn based on the available evidence. For atovaquone, the available data showed a lower maximum concentration and exposure. Finally, the maximum concentration of cycloguanil, the active metabolite of proguanil, was significantly lower, possibly compromising the efficacy.

**Conclusion:**

These findings suggest that reassessment of the dose of the artemisinin derivate and some components of ACT are necessary to ensure the highest possible efficacy of malaria treatment in pregnant women. However, for most components of ACT, data were insufficient and extensive research with larger sample sizes will be necessary to identify the exact influences of pregnancy on the pharmacokinetic properties of different artemisinin-based combinations. In addition, different clinical studies used diverse study designs with various reported relevant outcomes. Future pharmacokinetic studies could benefit from more uniform designs, in order to increase quality, robustness and effectiveness.

*Study registration*: CRD42015023756 (PROSPERO)

**Electronic supplementary material:**

The online version of this article (doi:10.1186/s12936-016-1160-6) contains supplementary material, which is available to authorized users.

## Background

Malaria infection during pregnancy remains an important public health problem with potential life-threatening risks for the pregnant woman, the foetus and the newborn child [1, 2]. According to a systematic review to assess the burden of malaria in pregnancy, approximately 25 million pregnant women are at risk of *Plasmodium falciparum* infection every year [3]. One in four women have evidence of placental infection at the time of delivery; of which a small fraction is encountered as an imported condition in non-endemic countries in migrants and travellers [4]. Imported cases of malaria in pregnancy are mainly *P. falciparum* acquired in sub-Saharan Africa [4]. Malaria in pregnancy is caused by all five species of Plasmodium infecting humans: *P. falciparum, P. vivax, P. ovale, P. malariae* and *P. knowlesi.* Most morbidity and mortality is caused by falciparum and vivax malaria. *Plasmodium knowlesi* malaria is endemic in parts of South East Asia and is relatively rare in pregnancy.

Pregnancy increases the risk of both falciparum and vivax malaria [3, 5]. The increased susceptibility has been attributed to broad hormonal and immunological changes that occur during pregnancy [5]. For *P. falciparum*, there is evidence that the increased susceptibility is due to the lack of immunity to antigens expressed only by parasites infecting pregnant women [5, 6]. It is unclear what causes the increased susceptibility for *P. vivax* malaria in pregnancy [5]. The prevalence of both falciparum and vivax malaria is higher in primigravidae than in non-pregnant women or multigravidae. As well, younger age is associated with higher risk for malaria in pregnancy [3, 5]. *Plasmodium falciparum* and *P. vivax* malaria are associated with maternal anaemia, lower birth weight and, in low-transmission areas, increased risks of spontaneous abortion, severe malaria and stillbirth [3, 7–10]. The increased burden of malaria in pregnancy has been attributed to higher parasite densities and the sequestration of *P. falciparum* infected erythrocytes (Pf-IEs) in the placenta [3, 5, 6, 11–13], resulting in placental changes including inflammation and disposition of pigment in fibrin or inflammatory cells, syncytial knotting and thickening of the trophoblastic basement membrane [5]. *Plasmodium vivax* however, does not cytoadhere in the placenta, but is associated with maternal anaemia and low birth weight [3].

In order to reduce the burden of malaria in pregnancy, the WHO recommends a three-pronged approach. Women are recommended to sleep under long-lasting insecticide-impregnated nets (LLINs) and to use intermittent preventive treatment (IPTp) with sulfadoxine-pyrimethamine (SP) when living in areas with a high to moderate stable transmission. The WHO emphasizes the importance of prompt diagnosis and effective case management of malaria infections [14]. Furthermore, all pregnant women should receive iron and folic acid supplementation as a part of routine antenatal care.

In the “Guidelines for the treatment of malaria” (Third edition, 2015), the WHO recommends [15] the use of an artemisinin-based combination therapy (ACT) for the treatment of uncomplicated falciparum malaria in the 2nd and 3rd trimester of pregnancy [16]. Over the past two decades multiple studies have been conducted to assess the efficacy and safety of ACT in the 2nd and 3rd trimester of pregnancy compared to other treatments [17–25]. However, pregnancy is known to cause physiologic and pharmacokinetic changes that might influence the efficacy of drugs. Different organ systems undergo changes which result in pharmacokinetic changes [26]. Pregnancy is associated with significant cardiovascular changes, especially in the first trimester of pregnancy. Cardiac output, stroke volume and heart rate increase, systemic and pulmonary vascular resistance decrease, as well as colloid osmotic pressure and haemoglobin concentration. This can increase the volume of distribution of hydrophilic substrates. Clinically, pregnant women sometimes need higher initial and subsequent dosage regimens, especially for hydrophilic drugs. In contrast, drugs that are bound to proteins or albumin can double the fraction of active pharmaceutical fraction. Respiratory changes in pregnancy include increased pulmonary vascularity, tidal volumes, minute volumes. In later stages of pregnancy, lung capacity may decrease because of the pressure from a big uterus on the diaphragm, causing alveolar collapse and atelectasis. In addition, pH of maternal blood may be increased, resulting in lower serum bicarbonate concentration and a lower buffering capacity. Furthermore, a rightward shift of the oxy-haemoglobin dissociation curve may affect protein binding of some drugs. Also the renal system is altered by pregnancy. In the first halve of pregnancy, the renal blood flow and GFR (glomerular filtration rate) is increased. Elimination rates can be higher for renal cleared drugs resulting in shorter half-lives. The bioavailability (e.g. Cmax, T1/2, Tmax) of oral anti-malarial drugs can also be changed by gastro-intestinal changes during pregnancy. There is delayed gastric emptying, and a longer small-bowel transit duration. These factors mainly influence single dose malaria treatments. Nausea and vomiting, common in early pregnancy, can also change the bioavailability of the anti-malarial drug caused by lower plasma concentrations. Also (sex) hormones during pregnancy increase or decrease the plasma concentrations of anti-malarial drugs. In the liver for example, CYP3A4 and cytochrome P450 are upregulated, resulting in a changed metabolism of CYP3A4 metabolized drugs (e.g. lumefantrine). Many other mechanisms have been described, however, the most important changes include increased maternal fat and total body water, decreased plasma protein concentrations, increased maternal blood volume and cardiac output and altered activity of hepatic drug-metabolizing enzymes [26].

Multiple studies have been conducted to study the influence of pregnancy on pharmacokinetic properties of ACT. However, due to small sample sizes it is often hard to draw strong conclusions based on these individual studies [27, 28].

## Objectives

The overall objective of this review was to summarize available evidence of the influence of pregnancy on the pharmacokinetic properties of different artemisinin-based combinations and the consequences these influences have on treatment dose and regime. The last review on this subject was published in 2009 [29]. The primary outcomes of interest for the analysis of the pharmacokinetics of the drugs in pregnancy were C_max_, T_max_, CL/F, V/F, t_1/2_ and total exposure (Area Under the Curve: AUC).

## Methods

This review was conducted in June 2015. The last search was conducted on 10 November 2015. Objectives and inclusion criteria were specified in advance and documented in a protocol. Recommendations made by the Preferred Reporting Items for Systematic Reviews and Meta-Analyses (PRISMA) group were followed [30]. This review was registered in advance in PROSPERO (International prospective register of systematic reviews). Registration number: CRD42015023756. The full methods section and search strategy are described in Additional file 1. An overview of ongoing or future trials is provided in Additional file 2. The costs of this literature study are not reported [31].

## Results

**Fig. 1:**
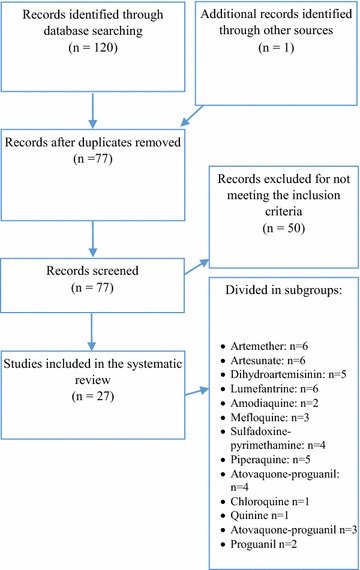
PRISMA flow diagram of study selection

The initial search yielded 121 records (Fig. 1: PRISMA flow diagram of study selection). 27 articles met the inclusion criteria and were included in the analysis (Table 1) [18, 19, 32–56]. The main study findings of the included trials can be found in Table 2.

**Table 1:** Overview and summary of included studies

Author (year)	Country *(time period)*	Type of study	Study population	Drug (dose)	Number of women	Pharmacokinetic analytic methodology	Pharmacokinetic variables	Remarks
Benjamin (2015) [56]	Papua New Guinea *(not reported)*	Clinical trial	Pregnant women with an EGA >14 weeks without severe malaria or other significant comorbidities and age-matched non-pregnant women without severe malaria and significant comorbidities	DHA-PPQ (7/58 mg/kg q.d. for 3 days) OR PPQ (1280 mg p.o. q.d. for 3 days) + SP (25 mg/kg once with first dose of PPQ)	32 pregnant women 33 non-pregnant women	Compartmental	CL/F, Vc/F, Vp/F, t1/2, AUC0-∞	
Valea (2014) [33]	Burkina Faso *(Sept 2008*–*Jan 2009)*	Clinical trial	Pregnant women in second and third trimester of pregnancy with uncomplicated *Plasmodium falciparum* mono-infection and matched non-pregnant women with *P. falciparum* infection	Mefloquine + Artesunate (8/3.6 mg/kg q.d. for 3 days)	24 pregnant women 24 non-pregnant women	Non-compartmental analysis	Total dose, Cmax, Cmax/dose, Tmax, CL/F, V/F, t1/2, AUC0-last, AUC0-∞, AUC-∞/dose	
Tarning (2013) [37]	Uganda *(Oct 2006* –*May 2009)*	Clinical trial	Pregnant women with uncomplicated *P. falciparum* infection with an EGA >13 weeks and non-pregnant women matched for history of fever, temp. >37.5 °C, smoking status and the level of parasitaemia	AL (80/480 mg p.o. b.i.d. for 3 days) + 200 ml milk tea OR Quinine (10 mg/kg p.o. t.i.d. for 7 days)	AL: 21 pregnant women Lumefantrine: 26 pregnant women 17 non-pregnant women Quinine: 21 pregnant women	Non-compartmental analysis	Total dose, Cmax, Cmax/dose, Tmax, CL/F, V/F, t1/2, AUC0-last, AUC0-∞, AUC-∞/dose, AUC72-last, AUC72-∞, day 7 concentration	Results for artemether and dihydroartemisinin are reported by Tarning (2012-2) [38] Nested in larger efficacy/safety study by Piola (2010) [19]
Adam (2012) [55]	Sudan *(Aug 2007*–*Feb 2008)*	Clinical trial	Pregnant women in 2nd and 3th trimester of pregnancy (EGA 15–40 weeks) with uncomplicated *P. falciparum* malaria and Hb > 7 g/dL. and age- and weight-matched non-pregnant women with uncomplicated *P. falciparum* malaria	DHA-PPQ (2.4/20 mg/kg q.d. for 3 days)	12 pregnant women 12 non-pregnant women	Non-compartmental analysis	Total dose, Cmax (after dose 1, 2 and 3), Tmax (after dose 1, 2 and 3), CL/F, V/F, T1/2, AUC0-last, AUC0-∞, AUC-∞/dose, AUC0-24, AUC24-48, AUC48-72, AUC72-∞, day 7 and 14 concentration	Based on the same clinical study as Hoglund (2012) [53]
Hoglund (2012) [53]	Sudan *(Aug 2007*–*Feb 2008)*	Clinical trial	Pregnant women in 2nd and 3rd trimester of pregnancy (EGA 15–40 weeks) with uncomplicated *P. falciparum* malaria and Hb > 7 g/dL. and age- and weight-matched non-pregnant women with uncomplicated *P. falciparum* malaria	DHA-PPQ (2.4/20 mg/kg q.d. for 3 days)	12 pregnant women 12 non-pregnant women	Compartmental analysis	Cmax, Tmax, t1/2, AUC 48-90, AUC0-90, day 7 and 28 concentration	Based on the same clinical study as Adam (2012) [55]
McGready (2012) [51]	Thailand *(April 2008*–*March 2008)*	Clinical trial	Pregnant women in second and third trimester of pregnancy (Ht > 25 %) with uncomplicated *P. falciparum* malaria and the same women post partum (3 months) without malaria	*Group 1* Artesunate (4 mg/kg) i.v. q.d. on day 0; artesunate (4 mg/kg) p.o q.d. on day 1–6 *Group 2* Artesunate (4 mg/kg) p.o. q.d. on day 0; artesunate (4 mg/kg) i.v. q.d. on day 1, artesunate (4 mg/kg) p.o. q.d. on day 2–6)	20 pregnant women *Group 1: 10 women* *Group 2: 10 women* 14 postpartum women	Non-compartmental analysis	Total dose, Cmax, Cmax/dose, Tmax, CL/F, V/F, t1/2, AUC0-∞, AUC0-∞/dose	
Tarning (2012-1) [34]	Thailand *(June 2008*–*Dec 2008)*	Clinical trial	Pregnant women in second and third trimester of pregnancy (Ht > 25 %) with uncomplicated *P. falciparum* malaria and matched non-pregnant women with *P. falciparum* malaria	DHA-PPQ (6.4/51.2 mg/kg p.o. q.d. for 3 days)	24 pregnant women 24 non-pregnant women	Compartmental analysis	Cmax, Tmax, CL/F, V/F, t1/2, AUC0-24, AUC0-92, day 7 and 28 concentration	Based on the same clinical study as Rijken (2011-2) [10]
Tarning (2012-2) [36]	Uganda *(March 2008*–*Sept 2008)*	Clinical trial	Pregnant women in second and third trimester of pregnancy (EGA > 13 weeks) with uncomplicated *P. falciparum* malaria	AL (80/480 mg p.o. b.i.d. for 3 days) + 200 ml milk tea	21 pregnant women	Compartmental analysis	Total dose, Cmax, Tmax, CL/F, V/F, t1/2, AUC60-last, AUC/dose	Nested in larger efficacy study by Piola (2010) [19]
Tarning (2012-3) [38]	Thailand *(Oct 2007*–*May 2008)*	Clinical trial	Pregnant women in second and third trimesters of pregnancy with acute *P. vivax* mono-infection and same women post partum (84–173 days) with (n = 7) or without (n = 12) *P. vivax* malaria	Amodiaquine (10 mg/kg p.o. q.d. for 3 days)	27 pregnant women 19 postpartum women	Compartmental analysis	Cmax, Tmax, t1/2, AUC-last	Based on the same clinical study as Rijken (2011-1) [40]
Morris (2011) [46]	DRC *(May 2007*–*Nov 2008)*	Clinical trial	Pregnant women in second (22–26 weeks) and third (32–36 weeks) trimester of pregnancy with asymptomatic *P. falciparum* parasitaemia (200–300,000 p/µL; Ht > 30 %) and same women post partum (3 months) with (n = 2) or without (n = 24) *P. falciparum* parasitaemia and non-pregnant female volunteers with asymptomatic *P. falciparum* parasitaemia (200–300,000 p/µL)	Artesunate (200 mg) p.o. q.d. on day 0 + SP (1725 mg) p.o. q.d. on day 1	26 pregnant women 26 postpartum women 25 non-pregnant women	Compartmental analysis	T1/2, CL/F, V/F	Based on the same clinical study as Onyamboko (2011) [41]
Onyamboko (2011) [41]	DRC *(May 2007*–*Nov 2008)*	Clinical trial	Pregnant women in second (22–26 weeks) and third (32–36 weeks) trimester of pregnancy with asymptomatic *P. falciparum* parasitaemia (200–300,000 p/µL; Ht > 30 %) and same women post partum (3 months) with (n = 2) or without (n = 24) *P. falciparum* parasitaemia and non-pregnant female volunteers with asymptomatic *P. falciparum* parasitaemia (200–300,000 p/µL)	Artesunate (200 mg) p.o. q.d. on day 0 + SP (1725 mg) p.o. q.d. on day 1	26 pregnant women 26 postpartum women 25 non-pregnant women	Non-compartmental analysis	Total dose, Cmax, Tmax, CL/F, V/F, t1/2, AUC0-∞	Based on the same clinical study as Morris (2011) [46]
Rijken (2011-1) [40]	Thailand *(Oct 2007*–*May 2008)*	Clinical trial	Pregnant women in second and third trimesters of pregnancy with acute *P. vivax* mono-infection and same women post partum (84–173 days) with (n = 7) or without (n = 12) *P. vivax* malaria	Amodiaquine (10 mg/kg p.o. q.d. for 3 days)	24 pregnant women 18 postpartum women	Non-compartmental analysis	Total dose, Cmax, Cmax/dose, Tmax, CL/F, V/F, t1/2, AUC0-last, AUC0-∞, AUC0-∞/dose, day 7 concentration	Based on the same clinical study as Tarning (2012-3) [38]
Rijken (2011-2) [39]	Thailand *(June 2008*–*Dec 2008)*	Clinical trial	Pregnant women in second and third trimester of pregnancy (Ht < 25 %) with uncomplicated *P. falciparum* malaria and matched non-pregnant women with *P. falciparum* malaria	DHA-PPQ (6.4/51.2 mg/kg p.o. q.d. for 3 days)	24 pregnant women 24 non-pregnant women	Non-compartmental analysis	Total dose, Cmax, Cmax/dose, CL/F, V/F, t1/2, AUC0-last, AUC0-∞, AUC0-∞/dose, AUC0-24, AUC24-48, AUC48-72, AUC72-∞, day 7, 14 and 28 concentration	Based on the same clinical study as Tarning 2012-1 [34]
Nyunt (2010) [42]	Mali, Mozambique, Sudan and Zambia *(not reported)*	Clinical trial	Pregnant women with an EGA 15–36 weeks without *P. falciparum* parasitaemia (Hb > 8 g/dL) and same women post partum (6–43 weeks) without *P. falciparum* parasitaemia and with Hb > 8 g/dL (Mali and Zambia)/postpartum women (>6 months) without *P. falciparum* parasitaemia and with Hb > 8 g/dL (Mozambique and Sudan) and matched non-pregnant women with acute uncomplicated falciparum malaria (Mozambique)	SP (1500/75 mg p.o. once)	97 pregnant women 77 postpartum women	Compartmental analysis	Total dose, Cmax, CL/F, V/F, t1/2, AUC0-∞, day 7 concentration	
Piola (2010) [19]	Uganda *(Oct 2006*–*May 2009)*	Clinical trial	Pregnant women with uncomplicated *P. falciparum* infection (<250,000 p/µL) with an EGA > 13 weeks and Hb > 7 g/dL	AL (80/480 mg p.o. b.i.d. for 3 days) + 200 ml milk	97 pregnant women	Non-compartmental analysis	Day 7 concentration	Based on the same clinical study as Tarning (2012-2) [36] and Tarning (2013) [37]
Karunajeewa (2009) [52]	Papua New Guinee *(Feb 2006*–*July 2006)*	Clinical trial	Pregnant women in second or third trimester of pregnancy without severe malaria (n = 17: *P. falciparum*/*P. vivax*/*P. malariae* parasitaemia; n = 13: no parasitaemia) and matched non-pregnant women (n = 9*: falciparum/vivax/malariae* parasitaemia; n = 21: no parasitaemia)	SP (1500/75 mg p.o. once) + Chloroquine (1350 mg p.o. q.d. for 3 days)	30 pregnant women 30 non-pregnant women	Compartmental analysis	CL/F, V/F, t1/2, AUC0-∞	
Tarning (2009) [35]	Thailand *(not reported)*	Clinical trial	Pregnant women in second or third trimester of pregnancy with uncomplicated symptomatic *P. falciparum* malaria	AL (80/480 mg p.o. b.i.d. for 3 days) + 200–250 ml chocolate milk (6–7 g fat)	103 pregnant women	Compartmental analysis	Total dose, CL/F, V/F, day 7 concentration	Nested in larger efficacy -/safety study by McGready (2008) [18]
McGready (2008) [18]	Thailand *(April 2004*–*Aug 2006)*	Clinical trial	Pregnant women in second or third trimester of pregnancy with acute uncomplicated *P. falciparum* malaria	AL (80/480 mg p.o. b.i.d. for 3 days) + 250 ml chocolate milk (7 g fat)	85 pregnant women	Non-compartmental analysis	Day 7 concentration	Based on the same clinical study as McGready (2006-2) [49] and Tarning (2009) [35]
Green (2007) [54]	Kenya *(1999*–*2000)*	Clinical trial	Primi- and secondi gravid women with uncomplicated singleton pregnancies with EGA 16–28 weeks and Hb > 8 g/dL without symptomatic malaria (n = 11: parasitaemic; n = 22: aparasitaemic) and same women post partum (2–3 months) without symptomatic malaria (n = 1: parasitaemic; n = 10: aparasitaemic)	SP (1500/75 mg p.o. once)	33 pregnant women *16 HIV*-*positive* *17 HIV*-*negative* 11 postpartum women *6 HIV*-*positive* *5 HIV*-*negative*	Compartmental analysis	CL/F, V/F, t1/2, AUC0-∞	
McGready (2006-1) [47]	Thailand *(Oct 2000*–*July 2001)*	Clinical trial	Pregnant women in second or third trimester of pregnancy with recrudescent uncomplicated *P. falciparum* malaria after 7-day quinine treatment and Ht > 25 %	Artesunate-AP (4/20/8 mg/kg p.o. q.d. for 3 days) + 200 ml chocolate milk (8 % fat)	24	Non-compartmental and compartmental analysis	Total dose, Cmax, Tmax, CL/F, V/F, t1/2, AUC48-72	
McGready (2006-2) [49]	Thailand *(April 2004*–*Aug 2004)*	Clinical trial	Pregnant women in the second and third trimester of pregnancy with recrudescent uncomplicated multi-drug resistant *P. falciparum* malaria after 7-day quinine treatment	AL (80/480 mg p.o. b.i.d. for 3 days) + 250 ml chocolate milk (7 g fat)	13 pregnant women	Non-compartmental and compartmental analysis	Total dose, Cmax, Tmax, CL/F, V/F, t1/2, AUC0-24, AUC 60-84, AUC/dose	
Na Bangchang (2005) [22]	Thailand *(Nov 2000–April 2001)*	Clinical trial	Pregnant women in third trimester of pregnancy with acute symptomatic *P. falciparum* mono-infection and Hb > 8 g/dL	AP (1000/400 mg p.o. q.d. for 3 days)	26 pregnant women	Compartmental analysis	Cmax, Tmax, AUC0-∞, PG-CG ratio	
McGready (2003-1) [23]	Thailand *(not reported)*	Clinical trial	Pregnant women in second or third trimester of pregnancy with recrudescent multi-drug resistant uncomplicated *P. falciparum* malaria after 7-day quinine treatment and Ht > 25 %	Artesunate-AP (4/20/8 mg/kg p.o. q.d. for 3 days) + 300 ml chocolate milk (8 % fat)	24 pregnant women	Non-compartmental and compartmental analysis	Cmax, Tmax, CL/F, V/F, AUC0-∞, AUC48-∞	
McGready (2003-2) [24]	Thailand *(not reported)*	Clinical trial	Healthy pregnant women with an EGA > 35 weeks and same women post partum (>2 months)	Proguanil (200 mg p.o. once)	45 pregnant women 45 postpartum women	Non-compartmental analysis	Total dose, Cmax (plasma and urine), 6 h concentration (plasma and urine)	
Na Bangchang (1994) [44]	Thailand *(Sept 1986*–*June 1988)*	Clinical trial	Pregnant women in first (n = 2) and third (n = 7) trimester of pregnancy with *P. falciparum* parasitaemia and non-pregnant women matched for age with *P. falciparum* parasitaemia	Mefloquine (15 mg/kg)	9 pregnant women 8 non-pregnant women	Compartmental analysis	Total dose, Cmax, Tmax, CL/F, V/F, t1/2	
Wangboonskul (1993) [32]	Thailand *(not reported)*	Clinical trial	Pregnant women in third trimester of pregnancy without *P. falciparum* malaria and same women post partum (>2 months) without *P. falciparum* malaria and healthy adult male volunteers without *P. falciparum* malaria^a^	Proguanil (200 mg p.o. once)	10 pregnant women 4 postpartum women 9 male patients^a^	Compartmental analysis	Cmax, Tmax, CL/F, t1/2, AUC	
Nosten (1990) [43]	Thailand *(not reported)*	Clinical trial	Pregnant women in third trimester of pregnancy	*Group 1:* Mefloquine (250 mg per week) *Group 2:* Mefloquine (125 mg per week)	20 pregnant women	Compartmental analysis	Cmax, Tmax, CL/F, t1/2, AUC	

*q.d.* once a day, *b.i.d.* twice a day, *t.i.d.* three times a day, *p.o.* per os (oral), *i.v.* intravenous, *AL* artemether-lumefantrine, *DHA-PPQ* dihydroartemisinin-piperaquine, *SP* sulfadoxine-pyrimethamine, *AP* atovaquone-proguanil, *PG* proguanil, *CG* cycloguanil, *C*_*max*_ maximum concentration after administration, *T*_*max*_ time to maximum concentration after administration, *CL/F* oral clearance, *V/F* apparent volume of distribution, *Vc/F* central volume of distribution, *Vp/F* peripheral volume of distribution, *T*_*1/2*_ half-life, *AUC* area under the curve (exposure), *Hb* haemoglobin, *Ht* haematocrit

^a^Data for male subjects were included from a previous study for comparison [60]

**Table 2:** Primary study outcomes per compound

Author (year)	Country (time period)	Population	Drug (dose)	Number of women	Result
Artemether					
Tarning (2013)	Uganda *(Oct 2006* –*May 2009)*	Pregnant women with uncomplicated *P. falciparum* infection with an EGA > 13 weeks and non-pregnant women matched for history of fever, temp. >37.5 °C, smoking status and level of parasitaemia	AL (80/480 mg p.o. b.i.d. for 3 days) + 200 ml milk tea	21 pregnant women	Estimated exposure to artemether and DHA was similar to that previously reported in pregnant Thai patients and lower than reported in adult non-pregnant Thai patients
Tarning (2012-2)	Uganda *(March 2008*–*Sept 2008)*	Pregnant women in second and third trimester of pregnancy (EGA > 13 weeks) with uncomplicated *P. falciparum* malaria	AL (80/480 mg p.o. b.i.d. for 3 days) + 200 ml milk tea	21 pregnant women	No statistically significant differences in pharmacokinetic properties between second and third trimester
McGready (2006-2)	Thailand *(April 2004*–*Aug 2004)*	Pregnant women in the second and third trimester of pregnancy with recrudescent uncomplicated multi-drug resistant *P. falciparum* malaria after 7-day quinine treatment	AL (80/480 mg p.o. b.i.d. for 3 days) + 250 ml chocolate milk (7 g fat)	13 pregnant women	No significant differences in the pharmacokinetic parameters of artemether and DHA between the second and third trimester. Comparison with data from literature showed a lower AUC and Cmax of artemether compared to male Thai patients and of DHA compared to non-pregnant patients

Author (year)	Country (time period)	Type of study	Population	Drug (dose)	Number of women	Pharmacokinetic analytic methodology	Pharmacokinetic variables	Remarks
Artesunate								
Kloprogge (2015)	Thailand *(April 2008*–*March 2009)*	Clinical trial	Pregnant women in second and third trimester of pregnancy (Ht > 25 %) with uncomplicated *P. falciparum* malaria and the same women post partum (3 months) without malaria	*Group 1* Artesunate (4 mg/kg) i.v. q.d. on day 0; artesunate (4 mg/kg) p.o q.d. on day 1–6 *Group 2* Artesunate (4 mg/kg) p.o. q.d. on day 0; artesunate (4 mg/kg) i.v. q.d. on day 1, artesunate (4 mg/kg) p.o. q.d. on day 2–6)	20 pregnant women *Group 1: 10 women* *Group 2: 10 women* 14 postpartum women	Compartmental analysis	AUC0-12, Cmax, Tmax, t1/2, CL/F, Vd/F	Based on the same clinical study as McGready (2012)
Valea (2014)	Burkina Faso *(Sept 2008*–*Jan 2009)*	Clinical trial	Pregnant women in second and third trimester of pregnancy with uncomplicated *P. falciparum* monoinfection and matched non-pregnant women with *P. falciparum* infection	Mefloquine + Artesunate (8/3.6 mg/kg q.d. for 3 days)	24 pregnant women 23 non-pregnant women	Noncompartmental analysis	Total dose, Cmax, Cmax/dose, Tmax, CL/F, V/F, t1/2, AUC0-last, AUC0-∞, AUC-∞/dose	
McGready (2012)	Thailand *(April 2008*–*March 2009)*	Clinical trial	Pregnant women in second and third trimester of pregnancy (Ht > 25 %) with uncomplicated *P. falciparum* malaria and the same women post partum (3 months) without malaria	*Group 1* Artesunate (4 mg/kg) i.v. q.d. on day 0; artesunate (4 mg/kg) p.o q.d. on day 1–6 *Group 2* Artesunate (4 mg/kg) p.o. q.d. on day 0; artesunate (4 mg/kg) i.v. q.d. on day 1, artesunate (4 mg/kg) p.o. q.d. on day 2–6)	20 pregnant women *Group 1: 10 women* *Group 2: 10 women* 14 postpartum women	Noncompartmental analysis	Total dose, Cmax, Cmax/dose, Tmax, CL/F, V/F, t1/2, AUC0-∞, AUC0-∞/dose	Based on the same clinical study as Kloprogge (2015)
Morris (2011)	DRC *(May 2007*–*Nov 2008)*	Clinical trial	Pregnant women in second (22–26 weeks) and third (32–36 weeks) trimester of pregnancy with asymptomatic *P. falciparum* parasitaemia (200–300,000 p/µL; Ht > 30 %) and same women post partum (3 months) with (n = 2) or without (n = 24) *P. falciparum* parasitaemia and non-pregnant female volunteers with asymptomatic *P. falciparum* parasitaemia (200–300,000 p/µL)	Artesunate (200 mg) p.o. q.d. on day 0 + SP (1725 mg) p.o. q.d. on day 1	26 pregnant women 26 postpartum women 25 non-pregnant women	Compartmental analysis	T1/2, CL/F, V/F	Based on the same clinical study as Onyamboko (2011)
Onyamboko (2011)	DRC *(May 2007*–*Nov 2008)*	Clinical trial	Pregnant women in second (22–26 weeks) and third (32–36 weeks) trimester of pregnancy with asymptomatic *P. falciparum* parasitaemia (200–300,000 p/µL; Ht > 30 %) and same women post partum (3 months) with (n = 2) or without (n = 24) *P. falciparum* parasitaemia and non-pregnant female volunteers with asymptomatic *P. falciparum* parasitaemia (200–300,000 p/µL)	Artesunate (200 mg) p.o. q.d. on day 0 + SP (1725 mg) p.o. q.d. on day 1	26 pregnant women 26 postpartum women 25 non-pregnant women	Noncompartmental analysis	Total dose, Cmax, Tmax, CL/F, V/F, t1/2, AUC0-∞	Based on the same clinical study as Morris (2011)
McGready (2006-1)	Thailand *(Oct 2000*–*July 2001)*	Clinical trial	Pregnant women in second or third trimester of pregnancy with recrudescent uncomplicated *P. falciparum* malaria after 7-day quinine treatment and Ht > 25 %	Artesunate-AP (4/20/8 mg/kg p.o. q.d. for 3 days) + 200 ml chocolate milk (8 % fat)	24	Noncompartmental and compartmental analysis	Total dose, Cmax, Tmax, CL/F, V/F, t1/2, AUC48-72	
Dihydroartemisinin								
Benjamin (2015)	Papua New Guinee (…)	Clinical trial	Pregnant women in second and third trimester of pregnancy (EGA > 14 weeks) and age-matched non-pregnant women with uncomplicated with malaria infection	*Group 1* DHA-PPQ (7/58 mg/kg p.o. q.d. for 3 days) *Group 2* PPQ (1280 mg p.o. q.d. for 3 days) + SP (25 mg/kg once)	32 pregnant women 33 non-pregnant women	Compartmental analysis	MTT, NN, CL/F, Vc/F, Q/F, Vp/F, t1/2, AUC0-∞	
Valea (2014)	Burkina Faso *(Sept 2008*–*Jan 2009)*	Clinical trial	Pregnant women in second and third trimester of pregnancy with uncomplicated *P. falciparum* monoinfection and matched non-pregnant women with *P. falciparum* infection	Mefloquine + Artesunate (8/3.6 mg/kg q.d. for 3 days)	24 pregnant women 23 non-pregnant women	Noncompartmental analysis	Total dose, Cmax, Cmax/dose, Tmax, CL/F, V/F, t1/2, AUC0-last, AUC0-∞, AUC-∞/dose	
McGready (2012)	Thailand *(April 2008*–*March 2009)*	Clinical trial	Pregnant women in second and third trimester of pregnancy (Ht > 25 %) with uncomplicated *P. falciparum* malaria and the same women post partum (3 months) without malaria	*Group 1* Artesunate (4 mg/kg) i.v. q.d. on day 0; artesunate (4 mg/kg) p.o q.d. on day 1–6 *Group 2* Artesunate (4 mg/kg) p.o. q.d. on day 0; artesunate (4 mg/kg) i.v. q.d. on day 1, artesunate (4 mg/kg) p.o. q.d. on day 2–6)	20 pregnant women *Group 1: 10 women* *Group 2: 10 women* 14 postpartum women	Noncompartmental analysis	Total dose, Cmax, Cmax/dose, Tmax, CL/F, V/F, t1/2, AUC0-∞, AUC0-∞/dose	Based on the same clinical study as Kloprogge (2015)
Tarning (2012-1)	Thailand *(June 2008*–*Dec 2008)*	Clinical trial	Pregnant women in second and third trimester of pregnancy (Ht > 25 %) with uncomplicated *P. falciparum* malaria and matched non-pregnant women with *P. falciparum* malaria	DHA-PPQ (6.4/51.2 mg/kg p.o. q.d. for 3 days)	24 pregnant women 24 non-pregnant women	Compartmental analysis	Cmax, Tmax, CL/F, V/F, t1/2, AUC0-24, AUC0-92, day 7 and 28 concentration	Based on the same clinical study as Rijken (2011-2)
Tarning (2012-2)	Uganda *(March 2008*–*Sept 2008)*	Clinical trial	Pregnant women in second and third trimester of pregnancy (EGA > 13 weeks) with uncomplicated *P. falciparum* malaria	AL (80/480 mg p.o. b.i.d. for 3 days) + 200 ml milk tea	21 pregnant women	Compartmental analysis	Total dose, Cmax, Tmax, CL/F, V/F, t1/2, AUC60-last, AUC/dose	Nested in larger efficacy study by Piola (2010)
Morris (2011)	DRC *(May 2007*–*Nov 2008)*	Clinical trial	Pregnant women in second (22–26 weeks) and third (32–36 weeks) trimester of pregnancy with asymptomatic *P. falciparum* parasitaemia (200–300,000 p/µL; Ht > 30 %) and same women post partum (3 months) with (n = 2) or without (n = 24) *P. falciparum* parasitaemia and non-pregnant female volunteers with asymptomatic *P. falciparum* parasitaemia (200–300,000 p/µL)	Artesunate (200 mg) p.o. q.d. on day 0 + SP (1725 mg) p.o. q.d. on day 1	26 pregnant women 26 postpartum women 25 non-pregnant women	Compartmental analysis	T1/2, CL/F, V/F	Based on the same clinical study as Onyamboko (2011)
Onyamboko (2011)	DRC *(May 2007*–*Nov 2008)*	Clinical trial	Pregnant women in second (22–26 weeks) and third (32–36 weeks) trimester of pregnancy with asymptomatic *P. falciparum* parasitaemia (200–300,000 p/µL; Ht > 30 %) and same women post partum (3 months) with (n = 2) or without (n = 24) *P. falciparum* parasitaemia and non-pregnant female volunteers with asymptomatic *P. falciparum* parasitaemia (200–300,000 p/µL)	Artesunate (200 mg) p.o. q.d. on day 0 + SP (1725 mg) p.o. q.d. on day 1	26 pregnant women 26 postpartum women 25 non-pregnant women	Noncompartmental analysis	Total dose, Cmax, Tmax, CL/F, V/F, t1/2, AUC0-∞	Based on the same clinical study as Morris (2011)
Rijken (2011-2)	Thailand *(June 2008*–*Dec 2008)*	Clinical trial	Pregnant women in second and third trimester of pregnancy (Ht < 25 %) with uncomplicated *P. falciparum* malaria and matched non-pregnant women with *P. falciparum* malaria	DHA-PPQ (6.4/51.2 mg/kg p.o. q.d. for 3 days)	24 pregnant women 24 non-pregnant women	Noncompartmental analysis	Total dose, Cmax, Cmax/dose, CL/F, V/F, t1/2, AUC0-last, AUC0-∞, AUC0-∞/dose, AUC0-24, AUC24-48, AUC48-72, AUC72-∞, day 7, 14 and 28 concentration	Based on the same clinical study as Tarning (2012-1)
McGready (2006-1)	Thailand *(Oct 2000*–*July 2001)*	Clinical trial	Pregnant women in second or third trimester of pregnancy with recrudescent uncomplicated *P. falciparum* malaria after 7-day quinine treatment and Ht > 25 %	Artesunate-AP (4/20/8 mg/kg p.o. q.d. for 3 days) + 200 ml chocolate milk (8 % fat)	24	Noncompartmental and compartmental analysis	Total dose, Cmax, Tmax, CL/F, V/F, t1/2, AUC48-72	
McGready (2006-2)	Thailand *(April 2004*–*Aug 2004)*	Clinical trial	Pregnant women in the second and third trimester of pregnancy with recrudescent uncomplicated multi-drug resistant *P. falciparum* malaria after 7-day quinine treatment	AL (80/480 mg p.o. b.i.d. for 3 days) + 250 ml chocolate milk (7 g fat)	13 pregnant women	Noncompartmental and compartmental analysis	Total dose, Cmax, Tmax, CL/F, V/F, t1/2, AUC0-24, AUC60-84, AUC/dose	
Lumefantrine								
Kloprogge (2013)	Uganda *(March 2008*–*Sept 2008)*	Clinical trial	Pregnant women in second and third trimester of pregnancy (EGA > 13 weeks) with uncomplicated *P. falciparum* malaria	AL (80/480 mg p.o. b.i.d. for 3 days) + 200 ml milk tea	115 pregnant women *26 venous samples* *89 capillary samples* 17 non-pregnant women (all venous samples)	Compartmental analysis	AUC0-∞, Cmax, T1/2, day 7 concentration	Nested in larger efficacy/safety study by Piola (2010)
Tarning (2013)	Uganda *(Oct 2006*–*May 2009)*	Clinical trial	Pregnant women with uncomplicated *P. falciparum* infection with an EGA > 13 weeks and non-pregnant women matched for history of fever, temp. >37.5 °C, smoking status and level of parasitaemia	AL (80/480 mg p.o. b.i.d. for 3 days) + 200 ml milk tea OR Quinine (10 mg/kg p.o. t.i.d. for 7 days)	AL: 21 pregnant women Lumefantrine: 26 pregnant women 17 non-pregnant women Quinine: 21 pregnant women	Noncompartmental analysis	Total dose, Cmax, Cmax/dose, Tmax, CL/F, V/F, t1/2, AUC0-last, AUC0-∞, AUC-∞/dose, AUC72-last, AUC72-∞, day 7 concentration	Results for artemether and dihydroartemisinin are reported by Tarning (2012-2). Nested in larger efficacy/safety study by Piola (2010)
Piola (2010)	Uganda *(Oct 2006*–*May 2009)*	Clinical trial	Pregnant women with uncomplicated *P. falciparum* infection (<250,000 p/µL) with an EGA > 13 weeks and Hb > 7 g/dL	AL (80/480 mg p.o. b.i.d. for 3 days) + 200 ml milk	97 pregnant women	Noncompartmental analysis	Day 7 concentration	Based on the same clinical study as Tarning (2012-2) and Tarning (2013)
Tarning (2009)	Thailand *(not reported)*	Clinical trial	Pregnant women in second or third trimester of pregnancy with uncomplicated symptomatic *P. falciparum* malaria	AL (80/480 mg p.o. b.i.d. for 3 days) + 200–250 ml chocolate milk (6-7 g fat)	103 pregnant women	Compartmental analysis	Total dose, CL/F, V/F, day 7 concentration	Nested in larger efficacy/safety study by McGready (2008)
McGready (2008)	Thailand *(April 2004*–*Aug 2006)*	Clinical trial	Pregnant women in second or third trimester of pregnancy with acute uncomplicated *P. falciparum* malaria	AL (80/480 mg p.o. b.i.d. for 3 days) + 250 ml chocolate milk (7 g fat)	85 pregnant women	Noncompartmental analysis	Day 7 concentration	Based on the same clinical study as McGready (2006-2) and Tarning (2009)
McGready (2006-2)	Thailand *(April 2004*–*Aug 2004)*	Clinical trial	Pregnant women in the second and third trimester of pregnancy with recrudescent uncomplicated multi-drug resistant *P. falciparum* malaria after 7-day quinine treatment	AL (80/480 mg p.o. b.i.d. for 3 days) + 250 ml chocolate milk (7 g fat)	13 pregnant women	Noncompartmental and compartmental analysis	Total dose, Cmax, Tmax, CL/F, V/F, t1/2, AUC0-24, AUC 60-84, AUC/dose	
Amodiaquine								
Tarning (2012-3)	Thailand *(Oct 2007*–*May 2008)*	Clinical trial	Pregnant women in second and third trimesters of pregnancy with acute *P. vivax* monoinfection and same women post partum (84–173 days) with (n = 7) or without (n = 12) *P. vivax* malaria	Amodiaquine (10 mg/kg p.o. q.d. for 3 days)	27 pregnant women 19 postpartum women	Compartmental analysis	Cmax, Tmax, t1/2, AUC-last	Based on the same clinical study as Rijken (2011-1)
Rijken (2011-1)	Thailand *(Oct 2007*–*May 2008)*	Clinical trial	Pregnant women in second and third trimesters of pregnancy with acute *P. vivax* monoinfection and same women post partum (84–173 days) with (n = 7) or without (n = 12) *P. vivax* malaria	Amodiaquine (10 mg/kg p.o. q.d. for 3 days)	24 pregnant women 18 postpartum women	Noncompartmental analysis	Total dose, Cmax, Cmax/dose, Tmax, CL/F, V/F, t1/2, AUC0-last, AUC0-∞, AUC0-∞/dose, day 7 concentration	Based on the same clinical study as Tarning (2012-3)
Mefloquine								
Valea (2014)	Burkina Faso *(Sept 2008*–*Jan 2009)*	Clinical trial	Pregnant women in second and third trimester of pregnancy with uncomplicated *P. falciparum* monoinfection and matched non-pregnant women with *P. falciparum* infection	Mefloquine + Artesunate (8/3.6 mg/kg q.d. for 3 days)	24 pregnant women 23 non-pregnant women	Noncompartmental analysis	Total dose, Cmax, Cmax/dose, Tmax, CL/F, V/F, t1/2, AUC0-last, AUC0-∞, AUC-∞/dose	
Na Bangchang (1994)	Thailand *(Sept 1986*–*June 1988)*	Clinical trial	Pregnant women in first (n = 2) and third (n = 7) trimester of pregnancy with *P. falciparum* parasitaemia and non-pregnant women matched for age with *P. falciparum* parasitaemia	Mefloquine (15 mg/kg)	9 pregnant women 8 non-pregnant women	Compartmental analysis	Total dose, Cmax, Tmax, CL/F, V/F, t1/2	
Nosten (1990)	Thailand *(not reported)*	Clinical trial	Pregnant women in third trimester of pregnancy	*Group 1:* Mefloquine (250 mg per week) *Group 2:* Mefloquine (125 mg per week)	20 pregnant women	Compartmental analysis	Cmax, Tmax, CL/F, t1/2, AUC	
Sulfadoxine-pyrimethamine								
Nyunt (2010)	Mali, Mozambique, Sudan and Zambia *(not reported)*	Clinical trial	Pregnant women with an EGA 15–36 weeks without *P. falciparum* parasitaemia (Hb > 8 g/dL) and same women post partum (6–43 weeks) without *P. falciparum* parasitaemia and with Hb > 8 g/dL (Mali and Zambia)/postpartum women (>6 months) without *P. falciparum* parasitaemia and with Hb > 8 g/dL (Mozambique and Sudan) and matched non-pregnant women with acute uncomplicated falciparum malaria (Mozambique)	SP (1500/75 mg p.o. once)	97 pregnant women 77 postpartum women	Compartmental analysis	Total dose, Cmax, CL/F, V/F, t1/2, AUC0-∞, day 7 concentration	
Karunajeewa (2009)	Papua New Guinee *(Feb 2006*–*July 2006)*	Clinical trial	Pregnant women in second or third trimester of pregnancy without severe malaria (n = 17: *P. falciparum*/vivax/*malariae* parasitaemia; n = 13: no parasitaemia) and matched non-pregnant women (n = 9: falciparum/vivax/*malariae* parasitaemia; n = 21: no parasitaemia)	SP (1500/75 mg p.o. once) + Chloroquine (1350 mg p.o. q.d. for 3 days)	30 pregnant women 30 non-pregnant women	Compartmental analysis	CL/F, V/F, t1/2, AUC0-∞	
Green (2007)	Kenya *(1999*–*2000)*	Clinical trial	Primi- and secundigravid women with uncomplicated singleton pregnancies with EGA 16–28 weeks and Hb > 8 g/dL without symptomatic malaria (n = 11: parasitaemic; n = 22: aparasitaemic) and same women post partum (2-3 months) without symptomatic malaria (n = 1: parasitaemic; n = 10: aparasitaemic)	SP (1500/75 mg p.o. once)	33 pregnant women *16 HIV*-*positive* *17 HIV*-*negative* 11 postpartum women *6 HIV*-*positive* *5 HIV*-*negative*	Compartmental analysis	CL/F, V/F, t1/2, AUC0-∞	
Piperaquine								
Benjamin (2015)	Papua New Guinee (*not reported*)	Clinical trial	Pregnant women in second and third trimester of pregnancy (EGA > 14 weeks) and age-matched non-pregnant women with uncomplicated with malaria infection	*Group 1* DHA-PPQ (7/58 mg/kg p.o. q.d. for 3 days) *Group 2* PPQ (1280 mg p.o. q.d. for 3 days) + SP (25 mg/kg once)	32 pregnant women 33 non-pregnant women	Compartmental analysis	MTT, NN, CL/F, Vc/F, Q/F, Vp/F, t1/2, AUC0-∞	
Adam (2012)	Sudan *(Aug 2007*–*Feb 2008)*	Clinical trail	Pregnant women in second and third trimester of pregnancy (EGA 15–40 weeks) with uncomplicated *P. falciparum* malaria and Hb > 7 g/dL. and age- and weight-matched non-pregnant women with uncomplicated *P. falciparum* malaria	DHA-PPQ (2.4/20 mg/kg q.d. for 3 days)	12 pregnant women 12 non-pregnant women	Noncompartmental analysis	Total dose, Cmax (after dose 1, 2 and 3), Tmax (after dose 1, 2 and 3), CL/F, V/F, T1/2, AUC0-last, AUC0-∞, AUC-∞/dose, AUC0-24, AUC24-48, AUC48-72, AUC72-∞, day 7 and 14 concentration	Based on the same clinical study as Hoglund (2012)
Hoglund (2012)	Sudan *(Aug 2007*–*Feb 2008)*	Clinical trial	Pregnant women in second and third trimester of pregnancy (EGA 15–40 weeks) with uncomplicated *P. falciparum* malaria and Hb > 7 g/dL. and age- and weight-matched non-pregnant women with uncomplicated *P. falciparum* malaria	DHA-PPQ (2.4/20 mg/kg q.d. for 3 days)	12 pregnant women 12 non-pregnant women	Compartmental analysis	Cmax, Tmax, t1/2, AUC 48-90, AUC0-90, day 7 and 28 concentration	Based on the same clinical study as Adam (2012)
Tarning (2012-1)	Thailand *(June 2008*–*Dec 2008)*	Clinical trial	Pregnant women in second and third trimester of pregnancy (Ht > 25 %) with uncomplicated *P. falciparum* malaria and matched non-pregnant women with *P. falciparum* malaria	DHA-PPQ (6.4/51.2 mg/kg p.o. q.d. for 3 days)	24 pregnant women 24 non-pregnant women	Compartmental analysis	Cmax, Tmax, CL/F, V/F, t1/2, AUC0-24, AUC0-92, day 7 and 28 concentration	Based on the same clinical study as Rijken (2011-2)
Rijken (2011-2)	Thailand *(June 2008*–*Dec 2008)*	Clinical trial	Pregnant women in second and third trimester of pregnancy (Ht < 25 %) with uncomplicated *P. falciparum* malaria and matched non-pregnant women with *P. falciparum* malaria	DHA-PPQ (6.4/51.2 mg/kg p.o. q.d. for 3 days)	24 pregnant women 24 non-pregnant women	Noncompartmental analysis	Total dose, Cmax, Cmax/dose, CL/F, V/F, t1/2, AUC0-last, AUC0-∞, AUC0-∞/dose, AUC0-24, AUC24-48, AUC48-72, AUC72-∞, day 7, 14 and 28 concentration	Based on the same clinical study as Tarning (2012-1)

The studies included were published between 1990 and 2015 and included a total of 829 pregnant and 377 non-pregnant patients. Articles were categorized by the drug that was evaluated. If more than one anti-malarial was administered, the study was included in both categories. In total, 34 patients received artemether (34 pregnant; 0 non-pregnant), 182 artesunate (AS) (94 pregnant; 88 non-pregnant), 48 DHA (56 pregnant, 57 non-pregnant), 341 lumefantrine (324 pregnant; 17 non-pregnant), 46 amodiaquine (27 pregnant, 19 non-pregnant), 85 mefloquine (53 pregnant; 32 non-pregnant), 279 SP (161 pregnant; 118 non-pregnant), 72 PPQ (68 pregnant; 69 non-pregnant) and 163 AP (105 pregnant, 58 non-pregnant).

### Quality assessment of the included studies

Details of the quality assessment are depicted in the table in Additional file 3. In summary, studies were given a median 23 points (range 16–29) out of 31. Most of the studies did not report how patients were selected and did not report the percentage of patients who agreed to participate. This could compromise the representativeness of the study population. There was no blinding of patients, researchers or statistical analysis. Overall, the quality of the included studies could be evaluated as moderate to good.

### Studies assessing the pharmacokinetics of ACT

The data sheet (excel file) of the included studies is provided as Additional file 4.

### Artemether and dihydroartemisinin

Two studies investigated the pharmacokinetic properties of artemether in pregnant women, following oral administration of artemether-lumefantrine (AL) (80/480 mg b.i.d. for 3 days) [36, 49]. Tarning et al. [36] studied 21 pregnant women in the second or third trimester of pregnancy with uncomplicated falciparum malaria. Both a compartmental (zero-order absorption followed by transit compartment absorption and a simultaneous one-compartment drug-metabolite model) and a noncompartmental analysis were performed. The first revealed no statistically significant covariates, indicating among others no difference between second and third trimester. Results obtained by noncompartmental analysis were used to compare the pharmacokinetic properties of pregnant women with the literature. Estimated exposure to artemether and its active metabolite DHA was similar to that reported in pregnant Thai patients [49] but lower than that reported in two studies in adult non-pregnant patients from Thailand [57, 58]. However, these results should be interpreted with caution since ethnicity might have an impact on the pharmacokinetic properties of these drugs.

McGready et al. [49] studied 13 women in the second and third trimester of pregnancy with recrudescent uncomplicated multi-drug resistant falciparum malaria after 7-day quinine treatment. A noncompartmental analysis revealed no significant differences in the pharmacokinetic parameters of artemether and DHA between the second and third trimester. Comparison with data from the literature showed a lower exposure (AUC) and maximum concentration (C_max_) of artemether compared to male Thai patients and of DHA compared to non-pregnant patients [57].

### Artesunate and dihydroartemisinin

Six studies investigated the pharmacokinetics after administration of AS [33, 41, 46, 47, 51, 59]. Three studies described the pharmacokinetic properties of AS as well as the pharmacokinetic properties of DHA, the active metabolite of AS, that is the principle source of anti-malarial activity after AS administration [33, 51]. McGready et al. [51] compared the pharmacokinetics of intravenous and oral AS in 20 women with malaria during pregnancy and 3 months post-partum without malaria. They found no significant differences in AS or DHA pharmacokinetics after intravenous administration. After oral administration, the exposure of AS and DHA (AUC) was significantly higher in pregnant women with malaria than in post-partum women without malaria. This can be explained by a higher bioavailability and lower oral clearance. The authors ascribed the differences to a disease related reduction in pre-systemic metabolism, as an active malaria infection tends to increase oral bioavailability, and not to the pregnancy. This assumption was supported by a decrease in DHA exposure at day 6 compared with day 0 and 1 in women with malaria but not in healthy women.

Kloprogge et al. [59] used the same data as McGready et al. but used a noncompartmental analysis to dissect and quantify the individual contributions of malaria and pregnancy to the altered pharmacokinetics. They found no effect of both malaria and pregnancy on pharmacokinetic properties of intravenous AS and DHA. However, their research showed opposite and independent effects for malaria (87 % increase) and pregnancy (23 % decrease) on the absolute oral bioavailability of artesunate. Both findings are in line with conclusions drawn by McGready et al. and ask for further dose optimization studies.

Valea et al. [33] studied the pharmacokinetics of AS and DHA in 24 pregnant women and 24 controls. They found a significantly lower oral clearance (CL/F) and higher exposure (AUC) of AS in pregnant women compared to the non-pregnant women. However, they did not find significant differences for DHA. It is important to notice that there was a significantly higher parasite density in the pregnant women’ group, possibly resulting in higher exposure, which might have masked the pregnancy-related effects.

Three other studies reported only the pharmacokinetic properties of DHA [41, 46, 47]. Onyamboko et al. [41] compared 26 pregnant women with malaria with the same women three months post-partum and with 25 non-pregnant parasitaemic controls. After a single dose of orally administred AS, there appeared to be no significant and clinically relevant differences in DHA pharmacokinetics between the women in pregnant and post-partum state. However, the exposure of DHA (AUC_free_) was significantly lower in pregnant women compared to non-pregnant controls (the authors used a 90 % confidence interval), which is consistent with a significantly increased clearance (CL/F) in the pregnant group. The authors described a couple of reasons for this apparent difference between pregnant women and non-pregnant controls and the absence of this difference between pregnant and post-partum women, namely that the physiological changes that occur during pregnancy might remain 3 months post-partum, that lactation influences pharmacokinetics, that most post-partum women were not parasitaemic and that a comparison in the same women mitigates the effects of potential other confounders.

The investigation by Morris et al. [46] is based on the same clinical study, but used a compartmental analysis (one compartmental analysis with mixed zero order, lagged first-order absorption for AS) instead of a non-compartmental analysis to describe the data. In the final model, pregnancy status was the only covariate that had a significant influence on DHA clearance, suggesting a faster clearance of DHA in pregnant women. Together with the data from Onyamboko et al., the authors conclude that this provides further evidence that higher doses of AS would be required in pregnant women. Another study by McGready et al. [47] from 2006 (n = 24) showed lower exposure (AUC) to DHA and higher rates of clearance and apparent volume of distribution in pregnant women compared to literature on non-pregnant women, although this should be interpreted with caution as differences in methodology of the studies might explain (part of) the differences [60].

### Dihydroartemisinin

Three articles report the pharmacokinetic properties of oral DHA [34, 39, 56]. Rijken et al. and Tarning et al. papers are based on one study into the pharmacokinetics of DHA after the oral administration of DHA-PQ (6.4 and 51.2 mg/kg p.o. q.d. for 3 days) [34, 39]. Rijken et al. [39] used a noncompartmental analysis to describe the pharmacokinetic properties in 24 pregnant women and 24 controls with acute falciparum malaria. They report no significant differences in total DHA exposure or maximum concentration between the two groups, although the DHA exposure after the first dose was significantly lower among the pregnant women, and there was seemingly a trend of lower exposure after the other doses. As well, there was a trend towards higher clearance in pregnant women, but this did not reach statistical significance. However, the authors warn for the potentially masking effect of the high inter-individual variability.

Using a mono-compartmental disposition model, Tarning et al. [34] report a 38 % lower total exposure to DHA in pregnant women compared to the controls (p = 0.001), consistent with a significantly higher apparent volume of distribution (p = 0.008) and clearance rate (p = 0.001). This could be explained by pregnancy related induction of hepatic glucuronidation enzymes resulting in an increased first-pass metabolism and accelerated clearance.

Benjamin et al. [56] report no differences in pharmacokinetic properties of DHA between pregnant and non-pregnant women without malaria after oral DHA-PQ (7 and 58 mg/kg q.d. for 3 days).

### Lumefantrine

The pharmacokinetic properties of lumefantrine were investigated in six studies [18, 19, 35, 37, 49, 61]. A study by McGready et al. [49] in 13 pregnant women with recrudescent falciparum malaria showed significantly lower lumefantrine AUC values in pregnant women than in non-pregnant patients from studies with uncomplicated malaria, caused by more rapid lumefantrine elimination in pregnant women. The large proportion of smokers among the non-pregnant patients made it difficult to draw strong conclusions on the cause of this difference. Also, they reported a proportion of 38 % of pregnant women with a day 7 lumefantrine concentration below 280 ng/ml, which is associated with high failure rates (49 %) [62].

The study by McGready et al. from 2006 was nested in a larger trial to assess the efficacy, safety, tolerability and pregnancy outcomes of AL in pregnant women [18]. Apart from these outcomes, the study by McGready et al. [18] from 2008 describes day 7 capillary plasma lumefantrine concentrations in 85 patients. In total, 35 % of patients had a day 7 capillary plasma concentration below 355 ng/ml (which corresponds with 280 ng/ml in venous plasma). All 21 (100 %) patients with levels over 600 ng/ml were cured, while patients with lower values had recrudescent infections (p < 0.001).

A third study nested in the trial mentioned above investigated the population pharmacokinetics of lumefantrine in 103 pregnant women with uncomplicated multidrug-resistant falciparum malaria [35]. Using a two-compartment model with first-order absorption and elimination, they predicted a 12 % odds increase in recrudescence and 7.2 % increase in apparent volume of distribution for each successive week of EGA on admission for pregnant women. Also, they showed a non-significant trend (p = 0.26) for the predicted day 7 median capillary lumefantrine concentrations to be lower in women with a recrudescent [n = 17; median concentration: 388 ng/ml (range 126–536)] or new infection [n = 21; median concentration: 377 ng/ml (range 136–1210)] compared to women who had no recrudescence [n = 65, median concentration: 427 ng/ml (range 135–1600)]. Based on different dose regime simulations using their final model, they recommend a 5-day regime instead of a 3-day regime to increase exposure to artemether and DHA and increase day 7 plasma lumefantrine concentration.

The study by Piola et al. [19] reports the data of a large efficacy study conducted in 304 pregnant women with uncomplicated falciparum malaria, of whom 152 received quinine en 152 received AL. For 97 of the women who received AL, the day 7 plasma lumefantrine concentration was available. 32 % of women had a lumefantrine concentration below 280 ng/ml (venous plasma). Also the reappearance of malaria was significantly associated with decreased plasma concentrations of lumefantrine [Reappearance: 422 ng/ml (range 15–3246) vs no reappearance: 240 ng/ml (range 123–454); p = 0.01].

Nested into this efficacy trial was a pharmacokinetic study, which compared pharmacokinetics of lumefantrine in pregnant (n = 26) and non-pregnant (n = 17) women [37]. This study by Tarning et al. showed no statistical difference in total lumefantrine exposure, apparent volume of distribution of elimination clearance between the two groups. T_max_ and T_1/2_ were significantly shorter in pregnant women. The day 7 concentration of lumefantrine was lower among pregnant women (488 ng/ml [range 30.7–3550] in pregnant women vs 720 [range 339–2150] in non-pregnant women), but this difference was not statistically significant (p = 0.128). Fifteen percent of the pregnant women had a day 7 lumefantrine venous plasma concentration below 280 ng/ml, while none of the women in the non-pregnant control group had day 7 lumefantrine venous plasma concentrations below 280 ng/ml. With this in view, the study suggested no significant correlation between week of EGA and drug exposure.

A third study, nested in the efficacy trial, was performed by Kloprogge et al. [61]. The same data as Tarning et al. was used, plus day 7 capillary lumefantrine concentrations of 89 pregnant women with *P. falciparum* malaria. Based on a transit-compartment absorption model followed by a two-compartment disposition mode, a 27 % lower day 7 lumefantrine concentration in pregnant women was found compared to non-pregnant women., caused by a 36.5 % decrease in intercompartmental clearance during pregnancy. Although this corresponded with a previous study in Thailand [49], the cure rate was higher in this study, possibly resulting from higher background immunity or less lumefantrine resistance in Uganda compared to Thailand.

### Amodiaquine

The pharmacokinetics of amodiaquine were investigated in two studies [38, 40]. Rijken et al. [40] reported no difference in pharmacokinetic parameters between pregnant women with *P. vivax* malaria (n = 24) and post-partum women (n = 19), except for the maximum concentration (C_max_) of desethylamodiaquine (DEAQ), the principle metabolite of amodiaquine, that was significantly lower in pregnant women (p = 0.019). However, because the difference was below 10 % and the total exposure to (desethyl)amodiaquine did not differ significantly, the clinical impact of this difference was considered limited. There was no significant difference in pharmacokinetic parameters between the 2nd and 3rd trimester of pregnancy. Also, there was no significant pharmacokinetic difference between post-partum women with *P. vivax* malaria and those without it, making it unlikely that a disease effect masked the differences between pregnant and post-partum women. Pregnant women with recurrent *P. vivax* malaria did have a significantly lower dose-normalized amodiaquine exposure than post-partum women (p = 0.036), suggesting that high drug exposure suppresses recurrent malaria.

Tarning et al. [38] did a compartmental analysis of the same data using a lagged first-order absorption with a two-compartment disposition model followed by a three-compartment disposition of desethylamodiaquine. They found a relatively small effect of age of amodiaquine clearance (1.36 % reduction per year), which might be related to an increased immunity in older patients and reflect reduced severity of the disease. Also, pregnant patients had a reduced absorption lag time (41.6 % decrease), possibly as a result of increased cardiac output resulting in an increased blood flow to the stomach and small intestine [63]. Neither pregnancy status nor estimated gestational age resulted in a clinically relevant impact on other pharmacokinetic parameters.

### Mefloquine

The pharmacokinetics of mefloquine in pregnancy have been investigated initially in the 90 s, and been re-examined recently [33, 43, 45]. Nosten et al. [43] investigated the pharmacokinetics in 20 pregnant women who received either 125 of 250 mg of oral mefloquine monotherapy. They compared their outcomes with the literature and found a higher oral clearance and a shorter terminal elimination half-life in pregnant women. There was no evidence of delayed absorption. Mean maximum concentrations of mefloquine in women who received 250 mg of AS were significantly lower than previously reported in six healthy Brazilian volunteers [64–76].

Na Bangchang et al. [45] compared nine pregnant women with eight non-pregnant women, all suffering from uncomplicated falciparum malaria, who received a single-dose treatment of mefloquine (15 mg/kg). They found a significantly lower maximum concentration of whole blood mefloquine (p = 0.015) and an increased total apparent volume of distribution (p = 0.046) in the pregnant women. The pregnant women also tended to have increased times to peak concentration (T_max_) compared to non-pregnant women. However, this difference was not statistically significant. Systematic clearance and terminal elimination half-life’s were similar in both groups. No correlation between EGA and apparent V/F, t_1/2_ of CL/F were seen.

Valea et al. [33] investigated the pharmacokinetic properties of mefloquine in combination with AS. They compared the pharmacokinetic parameters of 24 pregnant and 24 non-pregnant women with uncomplicated falciparum malaria, treated with a fixed-dose combination of oral mefloquine and AS (8/3.6 mg/kg per day for 3 days). They found very similar values for C_max_, T_max_ and V/F in pregnant and non-pregnant women. However, t_1/2_ was significantly longer in pregnant women than in non-pregnant women (390.2 vs 289.2 h; p < 0.001). Also, pregnant women tended to have longer clearance time and higher exposure to mefloquine, but these differences were not statistically significant. The exposure to carboxymefloquine (an inactive metabolite of mefloquine), however, was lower in pregnant women, consistent with a higher apparent volume of distribution and clearance. These findings suggest that the higher exposure to mefloquine may be a result of decreased carboxylation of mefloquine, although the increase in exposure to mefloquine was not completely proportional to the decrease in exposure to carboxymefloquine.

### Sulfadoxine-pyrimethamine

Three studies investigated the pharmacokinetic properties of SP [42, 52, 54]. Green et al. [54] used a one-compartmental analysis to compare the outcome of 33 pregnant (n = 11: parasitaemic; n = 22: aparasitaemic) and 11 post-partum women without symptomatic malaria (n = 1: parasitaemic; n = 10: aparasitaemic) treated with the standard single dose SP (1500/75 mg). Linear regression showed a significant effect of parity status on sulfadoxine half-life as well as on exposure (AUC). The half-life of sulfadoxine was significantly shorter (148 vs 256 h; p < 0.0001); exposure (AUC) was significantly lower (22,816 vs 40,106 µg/ml/h; p < 0.001); and the plasma clearance rate was significantly higher (65.9 vs 36.9 ml/h; p < 0.001) during pregnancy compared with the post-partum period. Pregnancy status showed no significant effect on the distribution volume. For pyrimethamine, none of the pharmacokinetic parameters differed significantly between the pregnant and post-partum women, although the median half-life and exposure (AUC) were lower, yet not significantly.

Karunajeewa et al. [52] compared the pharmacokinetic properties of SP in 30 pregnant and 30 age-matched non-pregnant women who received IPTp (SP 1500/75 mg once and chloroquine 1350 mg q.d. for 3 days). They found a significantly lower exposure (AUC) for sulfadoxine (33 %), for N-acetylsulfadoxine (a metabolite of sulfadoxine) and for pyrimethamine (32 %) in the pregnant group; which is in line with the significantly higher clearance rates for (N-acetyl)sulfadoxine and pyrimethamine found in this study. The total apparent volume of distribution was significantly higher in pregnant women for (N-acetyl)sulfadoxine and pyrimethamine. The terminal elimination rate of sulfadoxine and N-acetylsulfadoxine was significantly higher in pregnant women, while the terminal elimination rate of pyrimethamine was significantly lower in this group.

Nyunt et al. [42] investigated the pharmacokinetics of SP in Intermittent Preventive Treatment (IPTp) of malaria in 98 pregnant and 77 post-partum women from four African countries. Using a one-compartment model, they found significantly higher maximum concentrations of sulfadoxine in pregnant women, but lower exposure (AUC), faster clearance, smaller volume of distribution and shorter elimination half-lifes during pregnancy, all statistically significant. Day 7 concentrations of sulfadoxine did not differ significantly; however, after adjusting for potential covariates, they were significantly lower during pregnancy. Day 7 concentrations appeared to be lower in the third than in the second trimester, but this difference was not statistically significant. For pyrimethamine, drug exposure was higher during pregnancy, which is consistent with a significantly slower total clearance, longer elimination half-life and an apparently smaller distribution volume during pregnancy. As well, the unadjusted day 7 concentration of pyrimethamine was significantly higher in pregnant women. All pharmacokinetic parameters varied significantly between the study sites.

### Piperaquine

Five articles report the pharmacokinetic properties of PQ. Two studies conducted by Rijken et al. and Tarning et al. are based on one study of the pharmacokinetics of PQ after the oral administration of DHA-PPQ (6.4 and 51.2 mg/kg q.d. divided over 3 days) [34, 39]. The findings for DHA are described above. Rijken et al. [39] used a non-compartmental analysis to describe the pharmacokinetic properties of PPQ in 24 pregnant and 24 non-pregnant women with falciparum malaria. They found no significant difference in total PPQ exposure between the pregnant and non-pregnant women. However, the exposure in the first 72 h (AUC0-24 h; AUC24-48 h and AUC48-72 h) was significantly higher in the pregnant women; as well, the day 7 concentration was significantly elevated. The apparent volume of distribution was significantly smaller (602 vs 877 L/kg; p = 0.0057) and the terminal elimination half-life was significantly shorter (17.8 vs 25.6 days; p = 0.0023) in the pregnant group. No statistically significant difference was found in clearance rate. The C_max_ was elevated after each dose, but only significantly after the first two doses.

Tarning et al. [34] did a compartmental analysis with the same data, using a three-compartment disposition model with a 45 % higher elimination clearance and a 47 % increase in relative bioavailability in pregnant women compared with non-pregnant women. They found no effect of pregnancy on exposure of PPQ. However, the terminal elimination half-life was shorter (17.5 vs 24.0 days; p < 0.001), the apparent volume of distribution lower (529 vs 829 L/kg; p < 0.001) and the maximum concentration higher (291 vs 216 ng/ml; p = 0.035) in the pregnant women. The time to maximum concentration, clearance rate and day 7 and 28 concentration did not differ significantly between the two groups.

Adam et al. [55] reported a significantly higher exposure (AUC0-24; 1.8 vs 0.86 µg h/ml; p = 0.01) and a longer time to maximum concentration (T_max_; 4.00 vs 1.50; p = 0.02) after the first dose of DHA-PPQ (24/20 mg/kg q.d. for 3 days) in 12 pregnant women compared to 12 non-pregnant women with uncomplicated falciparum malaria. No other significant differences were observed, including no difference in total exposure (AUC0-∞). That notwithstanding, there was a trend towards higher maximum concentrations of PPQ and a shorter half-life in pregnant women, compared to non-pregnant women.

A compartmental analysis using a three-compartment disposition model with a transit-absorption model of the same data was performed by Hoglund et al. [53]. They found a significantly higher maximum concentration (C_max_; 185 vs 102 ng/ml; p = 0.021) and longer time to maximum concentration (T_max_; 30.7 vs 1.48 h; p = 0.018) in the 12 pregnant women compared to the 12 non-pregnant women. The terminal elimination half-life was shorter in the pregnant group (t_1/2_; 22.1 vs 25.7 days; p = 0.001). However, no significant differences in exposure to PPQ and day 7 and 28 concentrations were observed.

Benjamin et al. [56] reported a 33 % lower exposure (AUC; 23.721 vs 35.644 µg h/L; p < 0.001) in pregnant women compared to non-pregnant women, consistent with a significantly shorter half-life (t_1/2_; 382 vs 488 h; p < 0.001) and a higher clearance rate (CL/F; 73.5 vs 53.8 L/h; p < 0.001).

### Atovaquone-proguanil (AP)

The pharmacokinetic properties of proguanil (PG) were first studied in 1993 by Wangboonskul et al. [32]. The pharmacokinetic parameters of PG (pro-drug), cycloguanil (CG) (active metabolite) and 4-chlorophenylbiguanide (inactive metabolite) were compared in 10 healthy pregnant women, four post-partum women and nine male patients; all treated with PG (200 mg p.o. once) [77]. They found a significantly lower maximum concentration of CG in pregnant women (C_max_; 12.5 vs 28.4 (post-partum) and 39.3 (male) ng/ml; p < 0.05) and a significantly shorter terminal elimination half-life for PG (t_1/2_; 12.3 vs 17.1 (post-partum) and 16.1 (male) h; p < 0.01) in the pregnant group. The total exposure (AUC) of CG in pregnant women was approximately half of the exposure in male and post-partum patients. The mean ratio of the exposure of PG to CG was 18.0 in pregnancy, compared to 7.8 post-partum. The 4-chlorophenylbiguanide maximum concentration was lower in the pregnant women than in the post-partum and male patients, but not significantly. All these observations seemed to indicate an impaired conversion of pro-drug to (active) metabolite.

McGready et al. [50] investigated the pharmacokinetic properties of AS-AP (4/20/8 mg/kg p.o. q.d. for 3 days) in 24 pregnant patients with recrudescent multi-drug resistant uncomplicated malaria. A noncompartmental and compartmental analysis was performed. For atovaquone, PG and CG, they found a lower (corrected) maximum concentration (C_max_) and total exposure (AUC) in pregnant women compared with healthy volunteers from the same population [78] and compared with Thai children with malaria [79]. The terminal elimination half-life of atovaquone was significantly longer in the pregnant women. The non-compartmental analysis for PG showed 40 % lower maximum concentrations (C_max_) and exposure (AUC) in pregnant women with acute malaria than in non-pregnant healthy adults. The lower PG concentrations were attributed in the population pharmacokinetic assessment to increased apparent volume of distribution and clearance rate.

Another study by McGready et al. [48] investigated the influences of pregnancy on biotransformation of PG to CG. They found similar PG plasma concentrations 6 h after administration of a single dose of PG (200 mg p.o.) in 45 women during pregnancy and the same 45 women at least two months post partum. Plasma concentrations (corrected for dose) of CG 6 h after administration of PG were significantly lower during pregnancy than post partum (25.0 vs 37.4 ng/ml; p < 0.01). The ratio of plasma and urine PG and CG concentrations increased significantly in pregnancy. The urine concentration of PG and CG were significantly higher during pregnancy.

Na Bangchang et al. [44] studied the pharmacokinetics of atovaquone and PG in pregnant women from Thailand (n = 8) and Zambia (n = 18) treated with AP (1000/400 mg p.o. q.d. for 3 days) for uncomplicated falciparum malaria. They found no significant differences in any of the pharmacokinetic parameters of atovaquone, PG or CG between patients from Thailand and Zambia. Atovaquone was slowly absorbed and slowly eliminated with considerable variation among individuals. The maximum concentration (C_max_) and exposure (AUC) of atovaquone found in this study were approximately half of those found in other studies in healthy volunteers and Thai children with malaria [79, 80]. No significant differences were seen in the pharmacokinetics of PG. Both the maximum concentration (C_max_) and exposure (AUC) of CG were considerably lower in this study compared to other studies [79–81].

## Discussion

This systematic review synthesized and compiled data on ACT pharmacokinetics and dynamics during pregnancy, and the consequences thereof on treatment dose and regime. The previous systematic review on this subject was published in 2009 [29]. The present review encompasses 27 reports with a total of 829 pregnant and 377 non-pregnant women [18, 19, 32–55]. The number of trials that were found are rather limited and disproportional low in view of to the large number of pregnant women with malaria infections worldwide. This has several reasons, first of all, pregnant women are systematically excluded from clinical trials (because of the risk of the unborn child to be exposed to harmful effects). Furthermore, there is a limited research funding available for trials, especially pharmacokinetic studies. Funding organizations prefer phase III or randomized clinical trials over pharmacokinetic studies. The identified studies also differed in quality (see Additional file 3). However, in general, study quality was moderate to good, and the evidence is strong enough to draw conclusions regarding pharmacokinetic changes that are exhibited in pregnant women. Although current WHO guidelines strongly recommend to treat malaria in the first trimester with quinine (combined with clindamycine), these will not be discussed in this review which is focussed on ACT.

### Artemether, artesunate and dihydroartemisinin

Artemisinin-based compounds have been investigated in good quality studies. Two studies on the pharmacokinetic properties of artemether and its principle metabolite DHA suggest that the maximum concentration (C_max_) and exposure (AUC) of artemether and DHA following treatment with oral AL are lower in pregnant women than in non-pregnant patients. It also showed that there is no significant difference between the second and third trimester of pregnancy [36, 49]. However, since there has been no direct comparative trial, these results should be interpreted with caution. A larger sample size comparative trial should be conducted to statistically confirm or rule out any clinically important effect of pregnancy on pharmacokinetic parameters.

Although the outcome of the studies on the pharmacokinetic properties of AS seem conflicting, they provide substantial evidence that pregnancy induces clearance and thus leads to lower exposure to AS and DHA. While three studies found a higher DHA exposure [51] or no significant differences in DHA pharmacokinetics between the two groups [33, 41], other studies provided enough evidence to explain this apparent contradiction by the independent and opposite effect of malaria infection on pharmacokinetic properties [41, 46, 49, 59]. The higher bioavailability and exposure resulting from malarial infection in thought to have masked the pregnancy related effects on pharmacokinetics. This conclusion is worrying as pregnant women might be at risk of under dosing, resulting in lower cure rates and a more rapid development of resistance to DHA, and urgently calls for reassessment of the treatment dose of artesunate. The exact mechanisms are unfortunately currently unknown. A dose optimization study will be necessary to find the ideal dose of artesunate-containing ACT in pregnancy in order to augment clinical efficacy and ensure safety.

Pharmacokinetic properties after administration of oral DHA were investigated in two studies. A non-compartmental analysis of the data of the first study showed a trend of lower exposure. However, the differences were only statistically significant after the first dose, possibly because of small sample sizes and high inter-individual variability. The compartmental analysis of the same data showed 38 % lower total exposure and significantly higher clearance rates and apparent volume of distribution. The second study reported no differences in pharmacokinetic properties between pregnant and non-pregnant women. Of interest, the first study included women with falciparum malaria, while the parasitaemic rate of the participants was very low in the second study. Further studies with larger sample sizes of the effect of pregnancy on DHA pharmacokinetics are recommended.

### Lumefantrine

An important development in the past 5 years are that several controlled studies were published on lumefantrine. Studies investigating the pharmacokinetic properties of lumefantrine show a low day 7 concentration and large proportions of concentrations below values that are associated with high failure rates [18, 19, 35, 37, 49, 61]. One study also showed a significantly lower lumefantrine exposure in pregnant women [49], while another study showed significantly shorter T_max_ and T_1/2_, but no statistically significant differences in lumefantrine exposure [37]. Especially the lower day 7 concentrations are reason for concern and ask for more extensive research and reconsideration of the drug dose. Future studies should investigate whether drug exposure improves with a standard six-dose regimen of lumefantrine given over 5 days compared to the standard regimen of 3 days. Important to note is that still much remains unknown regarding the variable absorption of lumefantrine (which is a lipophilic compound) in the digestive tract affecting pharmacokinetic values. In the included trials in this review, the absorption of lumefantrine was maximized by providing fatty foods (e.g. milk). However, in daily clinical practice in endemic countries, it is completely unknown which percentage of (pregnant) women co-administer high-fat foods.

### Amodiaquine

The reported studies with oral amodiaquine for the treatment of vivax malaria in Thailand revealed no clinically relevant differences in pharmacokinetic parameters between pregnant and post-partum women. There was no significant difference between post-partum women with malaria and without malaria, making it unlikely that a disease effect masked possible differences. Therefore, no dosage adjustments are recommended.

### Mefloquine

Two studies dating from 1990 and 1994 report significantly lower maximum concentrations of mefloquine in pregnant women compared to healthy Brazilian volunteers [43, 64] and non-pregnant women [45]. In contrast to a recent study that showed similar values for C_max_ in pregnant and non-pregnant women with falciparum malaria and a non-significant trend towards longer clearance and higher exposure to mefloquine. In the first two studies, patients received one dose of oral AS in a low dose as prophylaxis [43] or in a higher dose as treatment for chloroquine-resistant falciparum malaria [45], while in the recent study a 3 days course of treatment of given [33]. These differences in drug regimes, besides other variables, might have an influence on the pharmacokinetic parameters. It was thought that altered plasma concentrations of mefloquine were caused by the increased volume of distribution. The results of the most recent trial [33] suggests that although the exposure was higher, the carboxymefloquine concentration was reduced (with simultaneous increase in V/F and removal of the metabolite). The reduced carboxylation might be partially responsible for the of the compound in pregnant women. Larger trials with comparative drug regimens, metabolite profiles and compartmental analysis and patient groups would thus be necessary to draw strong conclusions on the effect of pregnancy on the pharmacokinetic properties of mefloquine.

### Sulfadoxine-pyrimethamine

The reported studies with SP (used for IPTp) all show a significant lower exposure, higher clearance rates and shorter terminal half-life of sulfadoxine in pregnant women compared with post-partum women [42, 52, 54]. This suggests that higher doses of sulfadoxine would be beneficial for the efficacy of IPTp even when the parasites are fully susceptible to sulfa drug combinations.

No general conclusion can be drawn for the pyrimethamine pharmacokinetics, since a study in Kenya showed no differences in pharmacokinetic parameters between pregnant and post-partum women [54], while a study in Papua New Guinea (PNG) reported significantly lower exposure and higher clearance rates [52]. A study in four African countries described significantly higher exposure and lower clearance rates in pregnant women. Possible explanations could be the different rates of parasitaemia between the studies (0–43 %) and the concurrent administration of chloroquine in the study in PNG. Further (observational or interventional) research will be necessary to assess the influence of pregnancy on the pharmacokinetic properties of pyrimethamine. However, with resistance hampering its use, the prospects for an extended PK evaluation of sulfa drug combinations used for IPTp appear to be limited [82]. The search for suitable alternative drug (combinations) for IPTp is ongoing [83].

### Piperaquine

The reported studies suggest an influence of pregnancy on the pharmacokinetic properties of PQ. Four of the five studies show either an elevated exposure after the first dose [39, 55], a (significantly) higher C_max_ and shorter terminal elimination half-lifes [34, 39, 53, 55]. However, none of the studies showed a significantly higher total exposure, probably limiting the clinical consequences of the influence of pregnancy. Thereby, one study reported a significantly lower exposure in the pregnant group, a shorter half-life and a higher apparent volume of distribution. These discrepancies emphasize the need for further research.

### Atovaquone

The studies investigating atovaquone pharmacokinetics show a more than 50 % lower C_max_ and total exposure in pregnant women with falciparum malaria compared to healthy volunteers [44, 50]. Although these results should be interpreted with caution due to small sample sizes, higher dosing of atovaquone ought to be considered.

### Proguanil

The pharmacokinetic parameters of PG did not differ significantly in most studies, but the maximum concentration and terminal elimination half-life of the active metabolite of PG, CG, were significantly lower and shorter in pregnant women. Also, the PG–CG-ratio was significantly elevated in two studies, suggesting an impaired conversion of PG to CG. Since CG is mainly responsible for the therapeutic effect of PG, this might compromise the efficacy; and as well, the dose of the PG portion in the AP combination may have to be re-considered.

## Limitations

This systematic review is subject to several limitations. First, there is a considerable degree of heterogeneity in the outcomes and parameters that were reported in the articles. For example, time intervals for exposure differed significantly between the studies; which makes it more difficult to compare the outcomes of different studies. Also, different units and methods of measurement for the outcomes were used in different studies, complicating direct comparisons. Secondly, there was a large heterogeneity in the control groups. In some studies, the pregnant women acted as their own post-partum controls, with or without (symptomatic) malaria, while in other studies non-pregnant women or men were used, also with or without (symptomatic) malaria. In some studies no control group was available, so data were compared with literature values. There was a large heterogeneity between the pregnant women, especially regarding parasite density and gestational age estimates. Furthermore, the pharmacokinetic sampling schemes differed among studies, which might have had an effect on the outcome parameters. Last, the relatively small sample sizes of all studies made it difficult to statistically confirm differences in pharmacokinetic properties. For these reasons, a meta-analysis could unfortunately not be conducted.

## Conclusion

This systematic review suggests that reassessment of the dose of some components of ACT is necessary to ensure the highest possible efficacy of malaria treatment in pregnant women. Especially for artesunate, lumefantrine, sulfadoxine, atovaquone and proguanil evidence suggest that with current regimes, pregnant women are under-dosed. Evidence also indicates that the effect of pregnancy on amodiaquine and piperaquine is clinically not relevant and dose thus does not have to be changed. For artemether, dihydroartemisinin, lumefantrine, mefloquine and pyrimethamine more extensive research with larger sample sizes is needed to draw strong conclusions on the effect of pregnancy on the pharmacokinetic parameters of these components of ACT. Novel approaches such as sampling methods using lower blood volumes and minimal preparation (e.g. dried blood spots, high sensitive drug assays), and population pharmacokinetics analysis that are able to investigate the effect of variables such as age, pregnancy, simultaneously administered drugs, can generate solid data that can inform treatment of malaria in pregnancy [84].

## Additional files

10.1186/s12936-016-1160-6 Methods section. This additional file describes the full methods and search strategy.
10.1186/s12936-016-1160-6 Ongoing trials. This additional file summarized ongoing clinical trials on the subject.
10.1186/s12936-016-1160-6 Quality of included studies. This additional file shows the results of the quality assessment of the individual included studies.
10.1186/s12936-016-1160-6 Data sheet. This additional file shows the original data as extracted from the included studies.

## Abbreviations

AS: artesunate
AM: artemether
q.d.: once a day
b.i.d.: twice a day
t.i.d.: three times a day
p.o.: per os (oral)
i.v.: intravenous
AL: artemether-lumefantrine
DHA-PQ: dihydroartemisinin-piperaquine
SP: sulfadoxine-pyrimethamine
AP: atovaquone-proguanil
PG: proguanil
CG: cycloguanil
Cmax: maximum concentration after administration
Tmax: time to maximum concentration after administration
CL/F: oral clearance
V/F: apparent volume of distribution
Vc/F: central volume of distribution
Vp/F: peripheral volume of distribution
T1/2: half-life
AUC: area under the curve (exposure)
Hb: haemoglobin
Ht: haematocrit
Wks: weeks

## References

[1] Dellicour S, Tatem AJ, Guerra CA, Snow RW, ter Kuile FO (2010). Quantifying the number of pregnancies at risk of malaria in 2007: a demographic study. PLoS Med.

[2] Mayor A, Bardaji A, Macete E, Nhampossa T, Fonseca AM, Gonzalez R (2015). Changing trends in *P. falciparum* burden, immunity, and disease in pregnancy. N Engl J Med.

[3] Desai M, ter Kuile FO, Nosten F, McGready R, Asamoa K, Brabin B (2007). Epidemiology and burden of malaria in pregnancy. Lancet Infect Dis.

[4] Kaser AK, Arguin PM, Chiodini PL, Smith V, Delmont J, Jimenez BC (2015). Imported malaria in pregnant women: a retrospective pooled analysis. Travel Med Infect Dis.

[5] Mc LA, Ataide R, Simpson JA, Beeson JG, Fowkes FJ (2015). Malaria and immunity during pregnancy and postpartum: a tale of two species. Parasitology.

[6] Hviid L, Salanti A (2007). VAR2CSA and protective immunity against pregnancy-associated *Plasmodium falciparum* malaria. Parasitology.

[7] van Geertruyden JP, Thomas F, Erhart A, D’Alessandro U (2004). The contribution of malaria in pregnancy to perinatal mortality. Am J Trop Med Hyg.

[8] Tobon-Castano A, Solano MA, Sanchez LG, Trujillo SB (2011). [Intrauterine growth retardation, low birth weight and prematurity in neonates of pregnant women with malaria in Colombia](in Portuguese). Rev Soc Bras Med Trop.

[9] Poespoprodjo JR, Fobia W, Kenangalem E, Lampah DA, Warikar N, Seal A (2008). Adverse pregnancy outcomes in an area where multidrug-resistant *Plasmodium vivax* and *Plasmodium falciparum* infections are endemic. Clin Infect Dis.

[10] McGready R, Davison BB, Stepniewska K, Cho T, Shee H, Brockman A (2004). The effects of *Plasmodium falciparum* and *P. vivax* infections on placental histopathology in an area of low malaria transmission. Am J Trop Med Hyg.

[11] Umbers AJ, Aitken EH, Rogerson SJ (2011). Malaria in pregnancy: small babies, big problem. Trends Parasitol.

[12] Rogerson SJ (2010). Malaria in pregnancy and the newborn. Adv Exp Med Biol.

[13] Ruizendaal E, van Leeuwen E, Mens PF (2015). Peripheral and placental biomarkers in women with placental malaria: a systematic review. Biomark Med.

[14] WHO. Lives at risk: malaria in pregnancy. Geneva: World Health Organization. [http://www.who.int/features/2003/04b/en/]. Accessed 10 Nov 2015.

[15] WHO. Guidelines for the treatment of malaria. Second edition. Geneva: World Health Organization. [http://whqlibdoc.who.int/publications/2010/9789241547925_eng.pdf?ua=1]. Accessed 10 Nov 2015.

[16] WHO. Guidelines for the treatment of malaria. Geneva: World Health Organization. [http://whqlibdoc.who.int/publications/2006/9241546948_eng_full.pdf]. Accessed 10 Nov 2015.

[17] Kaye DK, Nshemerirwe R, Mutyaba TS, Ndeezi G (2008). A randomized clinical trial comparing safety, clinical and parasitological response to artemether-lumefantrine and chlorproguanil-dapsone in treatment of uncomplicated malaria in pregnancy in Mulago hospital, Uganda. J Infect Dev Ctries.

[18] McGready R, Tan SO, Ashley EA, Pimanpanarak M, Viladpai-Nguen J, Phaiphun L (2008). A randomised controlled trial of artemether-lumefantrine versus artesunate for uncomplicated *Plasmodium falciparum* treatment in pregnancy. PLoS Med.

[19] Piola P, Nabasumba C, Turyakira E, Dhorda M, Lindegardh N, Nyehangane D (2010). Efficacy and safety of artemether–lumefantrine compared with quinine in pregnant women with uncomplicated *Plasmodium falciparum* malaria: an open-label, randomised, non-inferiority trial. Lancet Infect Dis.

[20] Bounyasong S (2001). Randomized trial of artesunate and mefloquine in comparison with quinine sulfate to treat *P. falciparum* malaria pregnant women. J Med Assoc Thai.

[21] Kalilani L, Mofolo I, Chaponda M, Rogerson SJ, Alker AP, Kwiek JJ (2007). A randomized controlled pilot trial of azithromycin or artesunate added to sulfadoxine-pyrimethamine as treatment for malaria in pregnant women. PLoS One.

[22] McGready R, Ashley EA, Moo E, Cho T, Barends M, Hutagalung R (2005). A randomized comparison of artesunate-atovaquone-proguanil versus quinine in treatment for uncomplicated falciparum malaria during pregnancy. J Infect Dis.

[23] McGready R, Brockman A, Cho T, Cho D, van Vugt M, Luxemburger C (2000). Randomized comparison of mefloquine-artesunate versus quinine in the treatment of multidrug-resistant falciparum malaria in pregnancy. Trans R Soc Trop Med Hyg.

[24] Mutabingwa TK, Muze K, Ord R, Briceno M, Greenwood BM, Drakeley C (2009). Randomized trial of artesunate + amodiaquine, sulfadoxine-pyrimethamine + amodiaquine, chlorproguanal-dapsone and SP for malaria in pregnancy in Tanzania. PLoS One.

[25] Sowunmi A, Oduola AMJ, Ogundahunsi OA, Fehintola FA, Ilesanmi OA, Akinyinka OO (1998). Randomised trial of artemether versus artemether and mefloquine for the treatment of chloroquine/sufadoxine-pyrimethamine-resistant falciparum malaria during pregnancy. J Obstet Gynaecol.

[26] Costantine MM (2014). Physiologic and pharmacokinetic changes in pregnancy. Front Pharmacol.

[27] Visser BJ, van Vugt M, Grobusch MP (2014). Malaria: an update on current chemotherapy. Expert Opin Pharmacother.

[28] Visser BJ, Wieten RW, Kroon D, Nagel IM, Belard S, van Vugt M (2014). Efficacy and safety of artemisinin combination therapy (ACT) for non-falciparum malaria: a systematic review. Malar J.

[29] Wilby KJ, Ensom MH (2011). Pharmacokinetics of antimalarials in pregnancy: a systematic review. Clin Pharmacokinet.

[30] Liberati A, Altman DG, Tetzlaff J, Mulrow C, Gotzsche PC, Ioannidis JP (2009). The PRISMA statement for reporting systematic reviews and meta-analyses of studies that evaluate health care interventions: explanation and elaboration. PLoS Med.

[31] Visser BJ, Buijink AW, Grobusch MP (2014). Reporting of medical research costs. Improving transparency and reproducibility of medical research. Methods Inf Med.

[32] Wangboonskul J, White NJ, Nosten F, ter Kuile F, Moody RR, Taylor RB (1993). Single dose pharmacokinetics of proguanil and its metabolites in pregnancy. Eur J Clin Pharmacol.

[33] Valea I, Tinto H, Traore-Coulibaly M, Toe LC, Lindegardh N, Tarning J (2014). Pharmacokinetics of co-formulated mefloquine and artesunate in pregnant and non-pregnant women with uncomplicated *Plasmodium falciparum* infection in Burkina Faso. J Antimicrob Chemother.

[34] Tarning J, Rijken MJ, McGready R, Phyo AP, Hanpithakpong W, Day NP (2012). Population pharmacokinetics of dihydroartemisinin and piperaquine in pregnant and nonpregnant women with uncomplicated malaria. Antimicrob Agents Chemother.

[35] Tarning J, McGready R, Lindegardh N, Ashley EA, Pimanpanarak M, Kamanikom B (2009). Population pharmacokinetics of lumefantrine in pregnant women treated with artemether-lumefantrine for uncomplicated *Plasmodium falciparum* malaria. Antimicrob Agents Chemother.

[36] Tarning J, Kloprogge F, Piola P, Dhorda M, Muwanga S, Turyakira E (2012). Population pharmacokinetics of artemether and dihydroartemisinin in pregnant women with uncomplicated *Plasmodium falciparum* malaria in Uganda. Malar J.

[37] Tarning J, Kloprogge F, Dhorda M, Jullien V, Nosten F, White NJ (2013). Pharmacokinetic properties of artemether, dihydroartemisinin, lumefantrine, and quinine in pregnant women with uncomplicated *Plasmodium falciparum* malaria in Uganda. Antimicrob Agents Chemother.

[38] Tarning J, Chotsiri P, Jullien V, Rijken MJ, Bergstrand M, Cammas M (2012). Population pharmacokinetic and pharmacodynamic modeling of amodiaquine and desethylamodiaquine in women with *Plasmodium vivax* malaria during and after pregnancy. Antimicrob Agents Chemother.

[39] Rijken MJ, McGready R, Phyo AP, Lindegardh N, Tarning J, Laochan N (2011). Pharmacokinetics of dihydroartemisinin and piperaquine in pregnant and nonpregnant women with uncomplicated falciparum malaria. Antimicrob Agents Chemother.

[40] Rijken MJ, McGready R, Jullien V, Tarning J, Lindegardh N, Phyo AP (2011). Pharmacokinetics of amodiaquine and desethylamodiaquine in pregnant and postpartum women with *Plasmodium vivax* malaria. Antimicrob Agents Chemother.

[41] Onyamboko MA, Meshnick SR, Fleckenstein L, Koch MA, Atibu J, Lokomba V (2011). Pharmacokinetics and pharmacodynamics of artesunate and dihydroartemisinin following oral treatment in pregnant women with asymptomatic *Plasmodium falciparum* infections in Kinshasa DRC. Malar J.

[42] Nyunt MM, Adam I, Kayentao K, van Dijk J, Thuma P, Mauff K (2010). Pharmacokinetics of sulfadoxine and pyrimethamine in intermittent preventive treatment of malaria in pregnancy. Clin Pharmacol Ther.

[43] Nosten F, Karbwang J, White NJ, Honeymoon, Na Bangchang K, Bunnag D (1990). Mefloquine antimalarial prophylaxis in pregnancy: dose finding and pharmacokinetic study. Br J Clin Pharmacol.

[44] Na-Bangchang K, Manyando C, Ruengweerayut R, Kioy D, Mulenga M, Miller GB (2005). The pharmacokinetics and pharmacodynamics of atovaquone and proguanil for the treatment of uncomplicated falciparum malaria in third-trimester pregnant women. Eur J Clin Pharmacol.

[45] Na Bangchang K, Davis TM, Looareesuwan S, White NJ, Bunnag D, Karbwang J (1994). Mefloquine pharmacokinetics in pregnant women with acute falciparum malaria. Trans R Soc Trop Med Hyg.

[46] Morris CA, Onyamboko MA, Capparelli E, Koch MA, Atibu J, Lokomba V (2011). Population pharmacokinetics of artesunate and dihydroartemisinin in pregnant and non-pregnant women with malaria. Malar J.

[47] McGready R, Stepniewska K, Ward SA, Cho T, Gilveray G, Looareesuwan S (2006). Pharmacokinetics of dihydroartemisinin following oral artesunate treatment of pregnant women with acute uncomplicated falciparum malaria. Eur J Clin Pharmacol.

[48] McGready R, Stepniewska K, Seaton E, Cho T, Cho D, Ginsberg A (2003). Pregnancy and use of oral contraceptives reduces the biotransformation of proguanil to cycloguanil. Eur J Clin Pharmacol.

[49] McGready R, Stepniewska K, Lindegardh N, Ashley EA, La Y, Singhasivanon P (2006). The pharmacokinetics of artemether and lumefantrine in pregnant women with uncomplicated falciparum malaria. Eur J Clin Pharmacol.

[50] McGready R, Stepniewska K, Edstein MD, Cho T, Gilveray G, Looareesuwan S (2003). The pharmacokinetics of atovaquone and proguanil in pregnant women with acute falciparum malaria. Eur J Clin Pharmacol.

[51] McGready R, Phyo AP, Rijken MJ, Tarning J, Lindegardh N, Hanpithakpon W (2012). Artesunate/dihydroartemisinin pharmacokinetics in acute falciparum malaria in pregnancy: absorption, bioavailability, disposition and disease effects. Br J Clin Pharmacol.

[52] Karunajeewa HA, Salman S, Mueller I, Baiwog F, Gomorrai S, Law I (2009). Pharmacokinetic properties of sulfadoxine-pyrimethamine in pregnant women. Antimicrob Agents Chemother.

[53] Hoglund RM, Adam I, Hanpithakpong W, Ashton M, Lindegardh N, Day NP (2012). A population pharmacokinetic model of piperaquine in pregnant and non-pregnant women with uncomplicated *Plasmodium falciparum* malaria in Sudan. Malar J.

[54] Green MD, van Eijk AM, van Ter Kuile FO, Ayisi JG, Parise ME, Kager PA (2007). Pharmacokinetics of sulfadoxine-pyrimethamine in HIV-infected and uninfected pregnant women in Western Kenya. J Infect Dis.

[55] Adam I, Tarning J, Lindegardh N, Mahgoub H, McGready R, Nosten F (2012). Pharmacokinetics of piperaquine in pregnant women in Sudan with uncomplicated *Plasmodium falciparum* malaria. Am J Trop Med Hyg.

[56] Benjamin JM, Moore BR, Salman S, Page-Sharp M, Tawat S, Yadi G (2015). Population pharmacokinetics, tolerability, and safety of dihydroartemisinin-piperaquine and sulfadoxine-pyrimethamine-piperaquine in pregnant and nonpregnant Papua New Guinean women. Antimicrob Agents Chemother.

[57] Lefevre G, Looareesuwan S, Treeprasertsuk S, Krudsood S, Silachamroon U, Gathmann I (2001). A clinical and pharmacokinetic trial of six doses of artemether-lumefantrine for multidrug-resistant *Plasmodium falciparum* malaria in Thailand. Am J Trop Med Hyg.

[58] Karbwang J, Na-Bangchang K, Congpuong K, Thanavibul A, Wattanakoon Y, Molunto P (1998). Pharmacokinetics of oral artemether in Thai patients with uncomplicated falciparum malaria. Fundam Clin Pharmacol.

[59] Kloprogge F, McGready R, Phyo AP, Rijken MJ, Hanpithakpon W, Than HH (2015). Opposite malaria and pregnancy effect on oral bioavailability of artesunate—a population pharmacokinetic evaluation. Br J Clin Pharmacol.

[60] Newton P, Suputtamongkol Y, Teja-Isavadharm P, Pukrittayakamee S, Navaratnam V, Bates I (2000). Antimalarial bioavailability and disposition of artesunate in acute falciparum malaria. Antimicrob Agents Chemother.

[61] Kloprogge F, Piola P, Dhorda M, Muwanga S, Turyakira E, Apinan S (2013). Population pharmacokinetics of lumefantrine in pregnant and nonpregnant women with uncomplicated *Plasmodium falciparum* malaria in Uganda. CPT Pharmacometrics Syst Pharmacol.

[62] White NJ, van Vugt M, Ezzet F (1999). Clinical pharmacokinetics and pharmacodynamics and pharmacodynamics of artemether-lumefantrine. Clin Pharmacokinet.

[63] Little BB (1999). Pharmacokinetics during pregnancy: evidence-based maternal dose formulation. Obstet Gynecol.

[64] Schwartz DE, Weidekamm E, Mimica I, Heizmann P, Portmann R (1987). Multiple-dose pharmacokinetics of the antimalarial drug Fansimef (pyrimethamine + sulfadoxine + mefloquine) in healthy subjects. Chemotherapy.

[65] Schwartz DE, Eckert G, Hartmann D, Weber B, Richard-Lenoble D, Ekue JM (1982). Single dose kinetics of mefloquine in man. Plasma levels of the unchanged drug and of one of its metabolites. Chemotherapy.

[66] Riviere JH, Back DJ, Breckenridge AM, Howells RE (1985). The pharmacokinetics of mefloquine in man: lack of effect of mefloquine on antipyrine metabolism. Br J Clin Pharmacol.

[67] Mimica I, Fry W, Eckert G, Schwartz DE (1983). Multiple-dose kinetic study of mefloquine in healthy male volunteers. Chemotherapy.

[68] Mansor SM, Navaratnam V, Mohamad M, Hussein S, Kumar A, Jamaludin A (1989). Single dose kinetic study of the triple combination mefloquine/sulphadoxine/pyrimethamine (Fansimef) in healthy male volunteers. Br J Clin Pharmacol.

[69] Looareesuwan S, White NJ, Warrell DA, Forgo I, Dubach UG, Ranalder UB (1987). Studies of mefloquine bioavailability and kinetics using a stable isotope technique: a comparison of Thai patients with falciparum malaria and healthy Caucasian volunteers. Br J Clin Pharmacol.

[70] Karbwang J, Looareesuwan S, Back DJ, Migasana S, Bunnag D, Breckenridge AM (1988). Effect of oral contraceptive steroids on the clinical course of malaria infection and on the pharmacokinetics of mefloquine in Thai women. Bull World Health Organ.

[71] Karbwang J, Bunnag D, Breckenridge AM, Back DJ (1987). The pharmacokinetics of mefloquine when given alone or in combination with sulphadoxine and pyrimethamine in Thai male and female subjects. Eur J Clin Pharmacol.

[72] Karbwang J, Back DJ, Bunnag D, Breckenridge AM (1988). A comparison of the pharmacokinetics of mefloquine in healthy Thai volunteers and in Thai patients with falciparum malaria. Eur J Clin Pharmacol.

[73] Juma FD, Ogeto JO (1989). Mefloquine disposition in normals and in patients with severe *Plasmodium falciparum* malaria. Eur J Drug Metab Pharmacokinet.

[74] Franssen G, Rouveix B, Lebras J, Bauchet J, Verdier F, Michon C (1989). Divided-dose kinetics of mefloquine in man. Br J Clin Pharmacol.

[75] Desjardins RE, Pamplin CL, von Bredow J, Barry KG, Canfield CJ (1979). Kinetics of a new antimalarial, mefloquine. Clin Pharmacol Ther.

[76] de Souza JM, Heizmann P, Schwartz DE (1987). Single-dose kinetics of mefloquine in Brazilian male subjects. Bull World Health Organ.

[77] Wattanagoon Y, Taylor RB, Moody RR, Ochekpe NA, Looareesuwan S, White NJ (1987). Single dose pharmacokinetics of proguanil and its metabolites in healthy subjects. Br J Clin Pharmacol.

[78] Looareesuwan S, Viravan C, Webster HK, Kyle DE, Hutchinson DB, Canfield CJ (1996). Clinical studies of atovaquone, alone or in combination with other antimalarial drugs, for treatment of acute uncomplicated malaria in Thailand. Am J Trop Med Hyg.

[79] Sabchareon A, Attanath P, Phanuaksook P, Chanthavanich P, Poonpanich Y, Mookmanee D (1998). Efficacy and pharmacokinetics of atovaquone and proguanil in children with multidrug-resistant *Plasmodium falciparum* malaria. Trans R Soc Trop Med Hyg.

[80] van Vugt M, Edstein MD, Proux S, Lay K, Ooh M, Looareesuwan S (1999). Absence of an interaction between artesunate and atovaquone–proguanil. Eur J Clin Pharmacol.

[81] Helsby NA, Ward SA, Edwards G, Howells RE, Breckenridge AM (1990). The pharmacokinetics and activation of proguanil in man: consequences of variability in drug metabolism. Br J Clin Pharmacol.

[82] Chico RM, Cano J, Ariti C, Collier TJ, Chandramohan D, Roper C (2015). Influence of malaria transmission intensity and the 581G mutation on the efficacy of intermittent preventive treatment in pregnancy: systematic review and meta-analysis. Trop Med Int Health.

[83] Sicuri E, Fernandes S, Macete E, Gonzalez R, Mombo-Ngoma G, Massougbodgi A (2015). Economic evaluation of an alternative drug to sulfadoxine-pyrimethamine as intermittent preventive treatment of malaria in pregnancy. PLoS One.

[84] Davis TM. Pharmacokinetic studies of antimalarials: recent developments. Expert Rev Clin Pharmacol. 2015;1–3. doi:10.1586/17512433.2016.1108190. 10.1586/17512433.2016.1108190 26512938

