# Supplementary material for: The influence of pregnancy on the pharmacokinetic properties of artemisinin combination therapy (ACT): a systematic review
Source: Malar J. 2016 Feb 18;15:99. doi: 10.1186/s12936-016-1160-6 (PMC4757991; doi:10.1186/s12936-016-1160-6)
Supplement: Supplementary file 1 — 10.1186/s12936-016-1160-6 Methods section. This additional file describes the full methods and search strategy. [file 12936_2016_1160_MOESM1_ESM.doc]

**Additional file 1:** Full method section

The first and second author searched the electronic databases Ovid Medline (1946 to November 2015), Ovid Embase (1947 to November 2015), Cochrane Central Register of Controlled Trials (November 2015), Web of Science (1975 to November 2015), CINAHL Plus with Full Text (1937 to November 2015), African Index Medicus (1933 to November 2015), African Journals Online (AJOL) (from inception to November 2015), Google Scholar (without patents and citations), Biosis Previews (1993 to November 2015) and PubMed (non-MEDLINE citations) (1947 to November 2015) to identify studies investigating the pharmacokinetics of ACTs in pregnant women. Also, major trial registries were searched to identify ongoing or future trials (Additional File 2). The search strategy consisted of free-text words and subject headings related to pregnancy, malaria and seven different components of ACTs. The search strategy for PubMed is depicted below. The search strategy was not limited by study design or language. Only human studies were included. Unpublished data (e.g. conference or meeting abstracts) were excluded. The bibliographies of all identified articles were examined to identify additional studies that were not identified during the computerized search. Endnote X7.1 (Thomson Reuters) was used to manage, de-duplicate and screen the references for eligibility. Studies retrieved were eligible for inclusion if they satisfied all selection criteria. The study population consisted of pregnant females with malaria *(P. falciparum , P. ovale, P. vivax, P. malariae & P. knowlesi* ) of all age groups. Treatment consisted of an artemisinin derivate or artemisinin combination treatment. Furthermore, data was available to assess the pharmacokinetics of either artemether, artesunate, dihydroartemisinin (DHA), lumefantrine, mefloquine, amodiaquine, sulfadoxine-pyrimethamine (SP), piperaquine (PPQ) or atovaquone-proguanil (AP). Both studies performing a compartmental and a non-compartmental analysis were included in the review. Eligibility assessment of studies found was performed independently in an unblinded standardized manner by the first two authors. The first author extracted the following study characteristics: first author, year of publication, geographic location, time of the study, type of study, administered drug and dosing regimen, the pharmacokinetic sampling regimen, drug quantification method, pharmacokinetic analytic methodology, number of pregnant and non-pregnant women treated with the drug, population characteristics including age, weight and estimated gestational age (EGA), pharmacokinetic outcome measurements including total dose, C_max_, C_max_/dose, T_max_, oral plasma clearance rate (CL/F), apparent volume of distribution (V/F), t_1/2_, day 7, 14 and 28 concentrations and exposure (Area Under the Curve; AUC) over multiple time spans for both pregnant and non-pregnant women and conclusion of the articles. Data was double checked for all articles included in the qualitative synthesis by the second author. A validity assessment was performed by the first author using the Downs and Black checklist for measuring study quality [1]. Details are included in Additional file 3: Quality of included studies. In short, articles were scored for quality of reporting, external validity, internal validity (bias and confounding) and statistical power. Quality was classified with 0 (poor) to 31 points (excellent).

The included studies were separated in nine groups based on the type of drug administered. These nine groups were: artemether, artesunate, dihydroartemisinin (DHA), lumefantrine, mefloquine, amodiaquine, sulfadoxine-pyrimethamine (SP), piperaquine (PPQ) and atovaquone-proguanil (AP). Some studies were used in multiple groups. Within each group, results were compared, and a general conclusion was drawn. These groups represent the components of first line drug therapies recommended by the WHO for pregnant women in the second and third trimester for the treatment of uncomplicated *P. falciparum* treatment (artemether-lumefantrine, artesunate-amodiaquine, artesunate-mefloquine, artesunate-SP and DHA-PPQ), of drugs recommended for intermittent preventive treatment in pregnancy (IPTp) (SP) and as prophylaxis for travellers (AP) [2, 3]. In the first trimester of pregnancy, the WHO recommends the use of 7 days with quinine plus clindamycin (10mg/kg bw twice a day) for the treatment of uncomplicated *P. falciparum* malaria (“strong recommendation”). An ACT or oral artesunate + clindamycin is an acceptable alternative if quinine + clindamycin is not available or is ineffective. These drugs are not included in this systematic review.

PubMed Search strategy

1. "Malaria"[MeSH] OR "Plasmodium"[MeSH] OR "malaria"[tiab] OR "plasmodium"[tiab]

AND

1. "Pregnancy"[MeSH] OR "Pregnan*"[tiab]

AND

1. "pharmacokinetics"[sh] OR "pharmacokinetics"[MeSH] OR "pharmacokinetic*"[tiab] OR “pharmacokinetics*”[tiab]

AND

1. ("artemisinin*"[tiab] OR "artesunate*"[tiab] OR "artemether*"[tiab] OR "dihydroartemisinin*"[tiab])

*OR*

("lumefantrine"[Supplementary Concept] OR "artemether-lumefantrine combination"[Supplementary Concept] OR "lumefantrine*"[tiab])

*OR*

("Mefloquine"[Mesh] OR "Ro 21-5104"[Supplementary Concept] OR "mefloquine-sulfadoxine-pyrimethamine"[Supplementary Concept] OR "mefloquine*"[tiab] OR "Lariam"[tiab] OR "ASMQ"[tiab] OR "artesunate-mefloquine"[tiab])

*OR*

("Amodiaquine"[Mesh] OR "amodiaquine, artesunate drug combination"[Supplementary Concept] OR "amodiaquine*"[tiab] OR "AQ*"[tiab])

*OR*

(("sulfadoxine-pyrimethamine-artesunate"[Supplementary Concept] OR "fanasil, pyrimethamine drug combination"[Supplementary Concept]) OR "SP"[tiab] OR (("sulfadoxine*"[tiab] OR "sulphadoxine*"[tiab]) AND "pyrimethamine*"[tiab]))

*OR*

("piperaquine"[Supplementary Concept] OR "piperaquine*"[tiab])

*OR*

("atovaquone, proguanil drug combination"[Supplementary Concept] OR "Atovaquone"[Mesh] OR "atovaquone*"[tiab] OR "Proguanil"[Mesh] OR "proguanil*"[tiab] OR "malarone*"[tiab])

**References**:
