# Supplementary material for: The influence of pregnancy on the pharmacokinetic properties of artemisinin combination therapy (ACT): a systematic review
Source: Malar J. 2016 Feb 18;15:99. doi: 10.1186/s12936-016-1160-6 (PMC4757991; doi:10.1186/s12936-016-1160-6)
Supplement: Supplementary file 2 — 10.1186/s12936-016-1160-6 Ongoing trials. This additional file summarized ongoing clinical trials on the subject. [file 12936_2016_1160_MOESM2_ESM.docx]

**Additional file 2**: Ongoing trials

**Database:** Trial registries

**Search strategy:** 1^st^ of September 2015 (update 1^st^ of November 2015)

| **Name of study** | **Registration ID (year)**  **country** | **Status** | **Antimalarial(s) tested** |
| --- | --- | --- | --- |
| **Australia and New Zealands’s (ANZCTR)** |  |  |  |
| Measurement of the pharmacokinetic parameters of azithromycin in pregnant women to evaluate its potential use in preventing malaria in this population | ACTRN12607000442404 | Registered | Azithromycin |
| **Clinicaltrials.gov** |  |  |  |
| Pharmacokinetics of mefloquine-artesunate in pregnant women with uncomplicated Plasmodium falciparum infection | NCT01082718 (2010) | Withdrawn | Artesunate  Mefloquine |
| Antimalarial pharmacology in children and pregnant women in Uganda | NCT01717885 (2012)  Uganda | Recruiting | Not specified |
| Pharmacokinetics, efficacy, gametocyte carriage, birth outcomes following sulfadoxine-pyrimethamine Intermittent Presumptive Treatment in pregnant women | NCT00380146 | Unknown | Sulfadoxine  Pyrimethamine |
| Artesunate plus sulfadoxine-pyrimethamine pharmacokinetics, efficacy, gametocytes carriage and birth outcomes in pregnant women with malaria | NCT00331708 | Unknown | Artesunate  Sulfadoxine  Pyrimethamine |

| **Trial registry** | **Number of identified studies conducted in sub Saharan Africa** |
| --- | --- |
| Australia and New Zealand's (ANZCTR) (http://www.anzctr.org.au) | 1 |
| Brazilian Clinical Trials Registry (ReBec) (http://www.ensaiosclinicos.gov.br) | 0 |
| Chinese Clinical Trial Registry (ChiCTR) (http://www.chictr.org) | 0 |
| Clinical Research Information Service (CRiS), Republic of Korea (http://cris.cdc.go.kr) | 0 |
| Clinical Trials Registry - India (CTRI) (http://ctri.nic.in) | 0 |
| Cuban Public Registry of Clinical Trials(RPCEC) (http://registroclinico.sld.cu) | 0 |
| EU Clinical Trials Register (EU-CTR) (https://www.clinicaltrialsregister.eu/) | 0 |
| German Clinical Trials Register (DRKS) (http://www.drks.de) | 0 |
| IFPMA Register (http://clinicaltrials.ifpma.org/clinicaltrials/  no_cache/en/myportal/index.htm) | 0 |
| Iranian Registry of Clinical Trials (http://www.irct.ir/) | 0 |
| Japan's UMIN-CTR (http://www.umin.ac.jp/ctr/) | 0 |
| The Netherlands' Trialregister.nl | 0 |
| The United States' ClinicalTrials.gov | 4 |
| The International ISRCTN.org | 0 |
| Pan African Clinical Trial Registry (PACTR) (http://www.pactr.org/) | 0 |
| Sri Lanka Clinical Trials Registry (SLCTR) (http://www.slctr.lk/) | 0 |
| **Total** | 5 |
